# Enough Terror to Belong: The Nonlinear Association of Death Anxiety with Group Identification

**DOI:** 10.1155/2024/3699789

**Published:** 2024-05-21

**Authors:** Chao Li, Jianning Dang, Li Liu

**Affiliations:** Faculty of Psychology, Beijing Key Laboratory of Applied Experimental Psychology, Beijing Normal University, Beijing, China

## Abstract

Death anxiety is presumed to be positively associated with group identification; however, recent evidence of a null correlation between the two constructs raises questions regarding this assumption. In contrast to the traditional linear perspective, we proposed and tested a J-shaped curvilinear association that only death anxiety beyond a certain threshold predicts group identification. Using two-wave longitudinal data from the UK, study 1 (*N* = 1,402) revealed that only after reaching a moderate-to-high level could death anxiety measured during the COVID-19 pandemic positively predict later identification with the community, one's country, and all humanity. Furthermore, using World Values Survey data, study 2 (*N* = 56,871) found that death-related anxiety (i.e., worry about a terrorist attack) was only positively associated with perceived closeness to one's village, county, and country after reaching a moderate-to-high level. Our findings provide a novel insight into the process of managing terror and the replication failure of the mortality salience effect.

## 1. Introduction

Individuals are frequently exposed to various stimuli or events that remind them of their own mortality, such as violence, disease outbreaks, natural disasters, and terrorist attacks [1–4]. According to terror management theory (TMT) [5, 6], human awareness of the inevitability of death conflicts with the innate desire for survival, thereby inducing death anxiety, which is defined as emotional distress related to death and dying (e.g., uneasiness, worry, concern, and fear) [7–9]. Failure to effectively manage death anxiety is associated with a range of mental health disorders, including depression, psychological pain, hypochondriasis, obsessive-compulsive disorder, and medically unexplained symptoms [10–13].

Humans' instinctive efforts to cope with death anxiety exert a profound motivational effect on their thoughts and behaviors, particularly by fostering the development of elaborate defensive mechanisms [14, 15]. Defenses against death thoughts or anxiety can take two forms: *proximal* defenses refer to rational and literal counters to fear of death, such as denying one's vulnerability to death, whereas *distal* defenses entail experiential and symbolic responses by embedding oneself in an enduring and culturally meaningful system [16–18]. Distal defenses are believed to be more effective for reducing death-related concerns in the long run (e.g., [16]) and have thus become the focus of TMT research.

Group identification is among the most significant distal defenses [15, 19] and refers to perceiving oneself as a member of a specific social group and having a psychological connection to that group (e.g., a sense of belonging and attachment) [20, 21]. Individuals typically identify with groups with which they perceive themselves as having similarities, proximity, and a shared fate [22–24]. Belonging to a social group that is larger and longer-lasting than the individual self can provide symbolic immortality and a sense of death transcendence (e.g., [25, 26]).

Building on this rationale, some empirical research has linked death anxiety to the hallmarks of greater group identification, such as ethnocentrism [27] and prosocial behavior toward one's community [28]. However, other studies do not support this positive link. For instance, previous research has reported that death anxiety is not significantly associated with a preference for ingroups [29] (experiment 4), identification with nations or organizations [30, 31], or care for humans (humanism) [32]. This controversy inspires us to revisit the traditional linear view on the relationship between death anxiety and group identification.

We argue that the linear perspective appears overly simplistic because it ignores how different levels of death anxiety may distinctly influence experiential responses. Responses have been found to change from rational to experiential as affective intensity increases to relatively high levels [33, 34]. Accordingly, group identification, as an experiential reaction, may only arise when one's level of death anxiety is sufficiently high. Therefore, we posit that death anxiety might be linked to group identification in a nonlinear manner.

Despite the lack of direct evidence, research on self-regulation of stress may provide some clues for our assumption. Coping with stress requires self-regulation [35], involving two closely interacting systems: “cool” and “hot.” The cool system is cognitive, reflective, and hippocampus-based, whereas the hot system is emotional, reflexive, and amygdala-based [36, 37]. Notably, stress levels influence the hot–cool balance; at low-to-moderate levels, the cool system takes over in responding, whereas at higher levels, the hot system begins to dominate during processing [38, 39]. As for this research, death anxiety is stressful [40–42], and managing the fear of death requires self-regulation [43]. Rational and experiential systems coincide with cool and hot systems, respectively [44, 45]; therefore, the dual defenses against death anxiety echo the twofold system of self-regulation. As such, when one experiences low-to-moderate death anxiety, proximal defenses (cool system) may be responsible for mitigating anxiety; however, with moderate-to-high anxiety levels, group identification as a distal defense (hot system) may take over in handling existential concerns.

Thus, we hypothesize that the relationship between death anxiety and group identification follows a J-shaped pattern: death anxiety is unrelated to group identification when it is below a moderate level (i.e., threshold) and positively associated with group identification when it is above this threshold. Modeling nonlinear patterns has been shown to reveal some significant relationships or processes that would otherwise be ignored when only linear associations are examined [46–49]. In a sense, by adopting a nonlinear perspective, the present research is promising to refine the TMT tradition regarding the process of countering death anxiety via group identification.

To our knowledge, this research is the first to test the nonlinear association between death anxiety and group identification. In study 1, using two waves of longitudinal data collected from the United Kingdom (UK) during the initial stage of the COVID-19 pandemic lockdown, we investigated the J-shaped prospective effect of death anxiety on group identification. In study 2, to conceptually replicate the findings of study 1 on a global scale, we analyzed cross-sectional survey data from a large international dataset and examined the curvilinear association between worry about a terrorist attack, used as a proxy measure of death anxiety, and group identification, operationalized as perceived closeness to certain groups.

## 2. Study 1

In study 1, we sought to establish evidence for the nonlinear longitudinal effect of death anxiety on group identification. We used data from the COVID-19 Psychological Research Consortium (C19PRC; https://osf.io/v2zur/), in which both death anxiety and group identification were measured with multi-item psychometric scales. The C19PRC, an Internet-based survey initiated in March 2020, assesses the long-term effects of the COVID-19 pandemic on various factors such as psychological, social, economic, and political outcomes [50]. By June 2023, data from six waves had been collected from multiple countries using Qualtrics. In this study, we analyzed the first two waves of data collected in the UK (i.e., C19PRC-UKW1 and C19PRC-UKW2), which include all of this study's measures of interest.

### 2.1. Method

#### 2.1.1. Participants

C19PRC-UKW1 recruited a nationally representative sample (*N* = 2,025) of the UK population between March 23 and 28, 2020, and a subsample (*N* = 1,406) of those who participated in the follow-up survey (C19PRC-UKW2) approximately one month later. After removing respondents with missing data for the key variables of interest, the final sample comprised 1,402 respondents from the UK (*M*_age_ = 49.22, SD_age_ = 14.95, 47.79% female) who responded to both waves 1 and 2.  Table 1 shows the sociodemographic characteristics of the final sample. In addition, 31 respondents (2.21%) in the final sample received a COVID-19 diagnosis during the two waves.

#### 2.1.2. Measures

*(1) Death Anxiety*. Death anxiety was measured using the 17-item Death Anxiety Inventory [51]. Respondents indicated the extent to which they agreed with each item (e.g., “The sight of a corpse deeply shocks me,” “I find it difficult to accept the idea that it all finishes with death,” and “I find it really difficult to accept that I have to die”) on a five-point Likert scale ranging from 1 (totally disagree) to 5 (totally agree). We averaged all items to create the death anxiety index (*α*_wave1_ = .94, *α*_wave2_ = .94). Higher values indicated higher levels of death anxiety.

*(2) Group Identification*. Group identification was measured using the nine-item adapted Identification with All Humanity Scale [52]. Respondents indicated their identification with and concern for three groups: (1) people in their community, (2) people from the UK, and (3) all humans everywhere. For each group, respondents answered three statements (e.g., “How much do you identify with (feel a part of, feel love toward, have concern for) …?”; 1 = not at all, 5 = very much). We averaged the three statements for each group to create indices of identification with the community (*α*_wave1_ = .87, *α*_wave2_ = .88), the country (*α*_wave1_ = .84, *α*_wave2_ = .85), and all humans (*α*_wave1_ = .87, *α*_wave2_ = .88). Higher values indicated higher levels of group identification.

*(3) Covariates*. We incorporated psychological and sociodemographic variables that have been associated with death anxiety or group identification in previous studies (e.g., [1, 53, 54]), as the covariates assessed at wave 1. These variables include COVID-19 anxiety (i.e., “How anxious are you about the COVID-19 pandemic?”), age, sex, highest educational level, employment status, gross annual household income, UK birth or upbringing status, ethnicity, and political orientation.

### 2.2. Analytic Approach

We adopted two analytical approaches to test for the nonlinear prospective effect of death anxiety on group identification. First, we fit two regression models for each group identification indicator: a linear regression model (model L1) and a quadratic regression model (model Q1). Following prior studies (e.g., [49, 55]), in both models, we regressed each group identification indicator at wave 2 on the linear or squared term of death anxiety at wave 1, with controlling for group identification indicators at wave 1. Specifically, model L1 included the linear term of death anxiety at wave 1 as the predictor, while model Q1 included both the linear and quadratic (i.e., squared) terms of death anxiety at wave 1 as predictors. Moreover, to test the robustness of the results, the models were separately estimated without (models L1 and Q1) and with (models L2 and Q2) the covariates. To put it another way, we created the quadratic regression models by adding squared terms of death anxiety at wave 1 to the linear ones. The score for death anxiety at wave 1 was mean-centered to ensure orthogonality between the linear and squared terms. Significant squared terms and evidence that the quadratic models fit the data better than the linear models would indicate the presence of nonlinearity [56]. Moreover, positive squared terms would suggest a curve opening upward, whereas negative squared terms would suggest a curve opening downward. Therefore, significant positive squared terms would indicate the potential presence of a J-shaped relationship.

Second, to test whether the nonlinear relationship follows a J-shaped pattern, we additionally used a “two-line” approach [57], which has been adopted in previous studies as a valid method to evaluate the shape of nonlinear relationships (e.g., [49, 58, 59]). This approach estimates the location of a breakpoint of the predictor and regression slopes before (at lower predictor values) and after (at higher predictor values) the breakpoint. Thus, we tested segmented models [60]. A nonsignificant slope before the breakpoint, combined with a significant positive slope after the breakpoint, would indicate the presence of a J-shaped relationship.

### 2.3. Results

**Figure 1:**
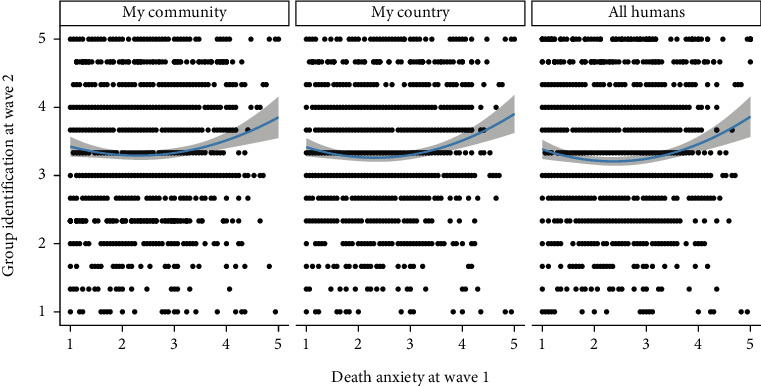
Death anxiety at wave 1 and group identification indicators at wave 2 in study 1. Gray shading indicates standard errors.

Table S1 shows descriptive statistics and zero-order correlations among the variables. Moreover, when controlling for group identification at wave 1, death anxiety at wave 1 was positively associated with identification with one's community (*r* = .07, *p* = .009), one's country (*r* = .07, *p* = .009), and all humans (*r* = .05, *p* = .045) at wave 2. Visual inspection of the data suggested that these associations might be curvilinear (Figure 1).

Next, we examined our hypothesized J-shaped relationship by conducting nonlinear association and J-shaped tests.

#### 2.3.1. Nonlinear Association Test: Linear versus Quadratic Models

**Figure 2:**
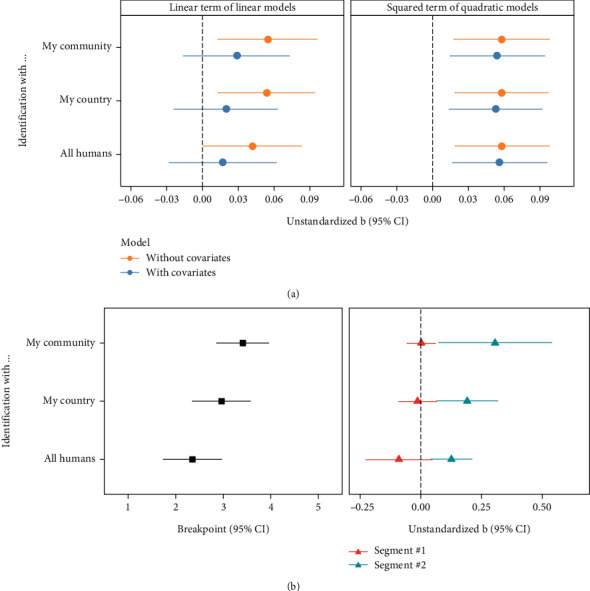
(a) Linear and quadratic models and (b) segmented models for each indicator of group identification at wave 2 in study 1. The linear term was mean-centered before computing the corresponding quadratic terms and entering the models. Segments 1 and 2 represent the slopes before and after the breakpoint, respectively. The 95% CIs for all effects are plotted.

We tested the linear and quadratic models for each group identification indicator (see Tables S2a–S2c for the full model results). Referring to previous research [49], we visualize the key results in Figure 2(a). The left panel of Figure 2(a) shows the regression coefficients of the linear effects of death anxiety. As depicted by orange dots, the linear effects of death anxiety on all group identification indicators were positive (ps < .045), with their 95% confidence intervals (CIs) excluding zero. However, as depicted by blue dots, these linear effects were not significant (ps > .212) when controlling for the covariates. The right panel of Figure 2(a) shows the regression coefficients of the quadratic effects of death anxiety. As depicted by orange dots, the squared terms were significantly positive for all group identification indicators (ps < .004). As depicted by blue dots, these terms remained significant after controlling for the covariates (ps < .007).

Further, the quadratic models fit the data better than the linear models, which were robust after controlling for the covariates (Table 2). Therefore, these findings provide evidence of the curvilinear lagged effect of death anxiety on group identification, as well as the possible presence of a J-shaped pattern.

#### 2.3.2. J-Shaped Curve Test: Segmented Models

Results from the segmented models (Figure 2(b); see Table S3 for the full model results) revealed a nonlinear lagged effect of death anxiety on group identification. Moreover, the nonlinear lagged effect was J-shaped. Specifically, for all three outcome indicators, the breakpoints ranged from 2.353 to 3.412; the slopes before the breakpoint (segment 1) were nonsignificant; however, the slopes after the breakpoint (segment 2) were significantly positive. Therefore, study 1 provides evidence for the hypothesized J-shaped and lagged effect of death anxiety on group identification (For supplementary purposes, we also explored the nonlinear relationship between death anxiety and group identification using only data from wave 1. These analyses (see *SM* for details) validated the curvilinear relationship: death anxiety did not show positive correlations with group identification indicators until reaching a moderate-to-high level. Before reaching a moderate level, death anxiety was not associated with any group identification indicators except for a negative link between low-to-moderate levels of death anxiety and identification with one's country. This deviation might be attributed to individuals with low-to-moderate levels of death anxiety cognitively distancing themselves from their country to alleviate concerns regarding the rapid increase in COVID-19 cases in the UK at the time [61]. However, with time, they might realize the inefficacy of this strategy, leading to a lack of predictive power of low-to-moderate death anxiety levels in national identification a month later, as death anxiety of these levels was not associated with identification with one's country at wave 2.).

## 3. Study 2

Study 2 is aimed at conceptually replicating the nonlinear pattern observed in study 1. Specifically, given that terrorist attacks have been shown to be closely linked to death-related thoughts and anxiety (e.g., [62–64]), we employed worry about a terrorist attack as a proxy measure of death anxiety. Moreover, to generalize our findings beyond a specific sample (e.g., the UK respondents in study 1), we used data from the World Values Survey (WVS), which is one of the largest and most widely used international surveys. The WVS data were primarily collected through face-to-face interviews conducted by the survey team at the respondents' place of residence. In this study, we analyzed data from wave 7 (2017–2022) [65], which is the only wave that includes all of this study's variables of interest.

### 3.1. Method

#### 3.1.1. Participants

After removing those with missing values for the key variables of interest, the final sample comprised 56,871 respondents (*M*_age_ = 43.01, SD_age_ = 16.54, 51.00% female) from 49 countries/regions. A list of the countries/regions and descriptive statistics by country/region is presented in Table S4.  Table 3 shows the sociodemographic characteristics of the final sample.

#### 3.1.2. Measures

*(1) Worry about a Terrorist Attack*. Worry about a terrorist attack was assessed using one item: “To what degree are you worried about a terrorist attack?” (1 = very much, 4 = not at all). The item was reverse coded, and higher values indicated higher levels of worry about a terrorist attack (*M* = 2.88, SD = 1.06).

*(2) Group Identification*. Group identification was measured using five indicators for perceived closeness to different groups, increasing in range from one's village to the world. Respondents were asked to indicate how close they felt to their (1) village, town, or city (*M* = 3.37, SD = 0.74); (2) county, region, or district (*M* = 3.21, SD = 0.80); (3) country (*M* = 3.25, SD = 0.80); (4) continent (*M* = 2.59, SD = 0.95); and (5) world (*M* = 2.51, SD = 0.98) on a four-point Likert scale ranging from 1 (very close) to 4 (not close at all). The items were reverse coded, and higher values indicated higher levels of identification.

*(3) Covariates*. Similar to study 1, we included age, sex, highest educational level, marital status, employment status, household income, subjective social status, native citizen, and political orientation as covariates.

### 3.2. Analytic Approach

The analytical strategy was similar to that of study 1, except that the data in this study were clustered in nature, with respondents being clustered within countries/regions. First, we examined the presence of nonlinear relationships by fitting linear regression models with or without the covariates (i.e., models L1 and L2) and quadratic regression models with or without the covariates (i.e., models Q1 and Q2). The score for worry about a terrorist attack was group mean-centered to make the linear and squared terms orthogonal. In addition, we estimated the random intercepts for each country/region in the multilevel models. Second, we used segmented multilevel models to check the shapes of the curves [66, 67].

### 3.3. Results

**Figure 3:**
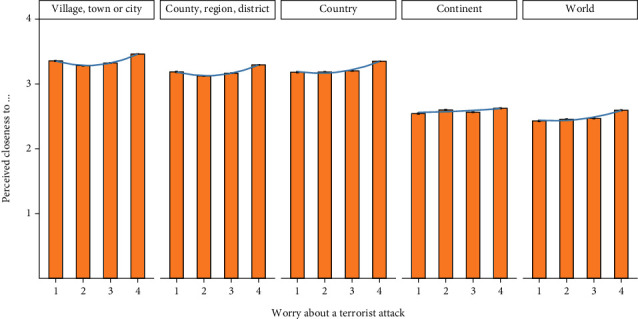
Worry about a terrorist attack and group identification indicators in study 2.

Table S5 shows the descriptive statistics and zero-order correlations among the variables. Overall, worry about a terrorist attack was positively associated with perceived closeness to one's (1) village, town, or city (*r* = .08, *p* < .001); (2) county, region, or district (*r* = .07, *p* < .001); (3) country (*r* = .09, *p* < .001); (4) continent (*r* = .02, *p* < .001); and (5) world (*r* = .06, *p* < .001) in all 49 countries/regions. However, a visual inspection of the data suggested that these associations might not be perfectly linear (Figure 3).

Similar to study 1, we employed nonlinear association and J-shaped tests to examine our hypothesis.

#### 3.3.1. Nonlinear Association Test: Linear versus Quadratic Models

**Figure 4:**
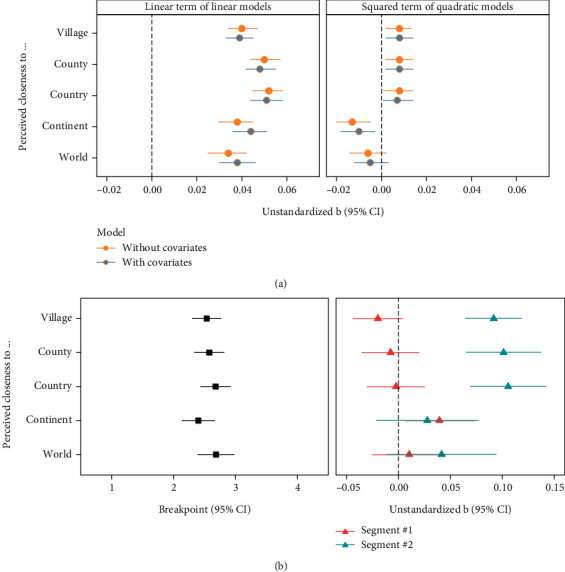
(a) Linear and quadratic models and (b) segmented models for each indicator of group identification in study 2. The linear term was group-mean-centered before computing the corresponding quadratic terms and entering the models. Segments 1 and 2 represent the slopes before and after the breakpoint, respectively. The 95% CIs for all effects are plotted.

We examined the nonlinear relationship between worry about a terrorist attack and group identification (see Tables S6a–S6e for the full model results). We visualize the key results in Figure 4(a). The left panel of Figure 4(a) shows the regression coefficients of the linear effects of worry about a terrorist attack. As depicted by orange dots, the linear effects of worry about a terrorist attack on all group identification indicators were positive (ps < .001), with their 95% CIs excluding zero. In addition, as depicted by blue dots, these effects remained significant after controlling for the covariates (ps < .001).

The right panel of Figure 4(a) shows the regression coefficients of the quadratic effects of worry about a terrorist attack. For perceived closeness to respondents' (1) village, town, or city (ps < .011); (2) county, region, or district (ps < .011); and (3) country (ps < .019), the quadratic terms of worry about a terrorist attack were significantly positive (as depicted by orange dots), even after controlling for the covariates (as depicted by blue dots). However, the quadratic terms of worry about a terrorist attack were significantly negative for perceived closeness to one's continent (ps < .005) and not significant for perceived closeness to the world (ps > .121), regardless of the presence of covariates.

Further, likelihood ratio tests (Table 2) revealed that the quadratic models fit the data better than the linear models without or with the covariates, except for perceived closeness to the world. These findings suggest nonlinear relationships between death anxiety (i.e., worry about a terrorist attack) and identification with one's home village, county, country, or continent, but not the world. Additionally, when considering the shape of nonlinear relationships, the hypothesized J-shaped pattern was present for identification with one's home village, county, and country, but not for identification with one's continent or the world.

#### 3.3.2. J-Shaped Curve Test: Segmented Models

Results from the segmented models (Figure 4(b); see Table S7 for the full model results) showed a curvilinear relationship between worry about a terrorist attack and perceived closeness to respondents' (1) village, town, or city; (2) county, region, or district; (3) country; and (4) continent, but not the world. Moreover, the nonlinear relationships between worry about a terrorist attack and perceived closeness to one's (1) village, town, or city; (2) county, region, or district; and (3) country were J-shaped. Specifically, for the three outcome indicators, the breakpoints were approximately 2.5 (ranging from 2.401 to 2.686); the slopes before the breakpoint (segment 1) were nonsignificant, whereas the slopes after the breakpoint (segment 2) were significantly positive. However, for perceived closeness to one's continent, the nonlinear relationship followed a flattening pattern, in which the slope was significantly positive before the breakpoint, but nonsignificant after. Thus, the results of study 2 indicate a J-shaped association between worry about a terrorist attack and perceived closeness to groups (i.e., village, county, and country). However, this was not the case for perceived closeness to the continent or world.

## 4. Discussion

The current research sought to explore the relationship between death anxiety and group identification from a nonlinear perspective. Research on self-regulation of stress indicates that varying levels of stress influence the balance between rational (cool) and experiential (hot) systems [38, 39, 44, 45]; hence, we posited that group identification as an experiential response may occur only once death anxiety reaches a moderate-to-high level. Accordingly, the current research examined the potential J-shaped curvilinear association between death anxiety and group identification.

To our knowledge, the present research offers the first empirical evidence of a curvilinear association between death anxiety and group identification. By analyzing longitudinal data from the UK respondents during the COVID-19 pandemic, study 1 indicates that only when death anxiety reaches a moderate-to-high level does it positively predict later identification with one's community and country, as well as all humans. Study 2, using data from 49 countries/regions (*N* = 56, 871), shows that, for individuals with moderate-to-high (but not low-to-moderate) levels of death-related anxiety, namely, worry about terrorist attacks, increased worry is linked to greater closeness to one's village, county, and country.

Interestingly, contrary to the hypothesized J-shaped relationship, study 2 found that even low levels of worry about terrorism correspond to identification with one's home continent and the world. This might be because countries have been increasingly cooperating closely on the continent (e.g., Europe) [68] and worldwide (e.g., [69]) levels to tackle terrorism. Thus, worry about terrorist attacks, even at low levels, may remind respondents of cooperation between countries across borders. Regarding the slight increase in identification with one's continent at high levels of worry, we speculate that when worry surpasses a certain threshold, people may regard citizens from different continents as sharing a common fate and turn to identifying with humanity as a whole. Certainly, these possibilities require further investigation.

The current research contributes to the existing research on TMT. On the one hand, it underscores the importance of death anxiety strength in predicting defensive mechanisms. Death anxiety, a universal emotional experience, is associated with various mental health disorders [70]. Psychologists have long been intrigued by how humans respond to death-related fear. One relevant influential theory is TMT, which identifies belonging to groups as a significant defense to alleviate death anxiety. However, possibly because of insufficient dialogue with emotion researchers [29], previous TMT investigators have overlooked the key role of emotional intensity in adopting coping strategies [71, 72]. Our research addresses this gap by exploring the distinct roles that different levels of death anxiety play in predicting group identification.

On the other hand, our research encourages a reevaluation of the psychological processes involved in managing terror. TMT indicates that proximal and distal defenses occur in a temporal sequence, regardless of the intensity of death anxiety. According to this logic, distal defenses such as group identification are enhanced as death anxiety increases. The J-shaped curvilinear relationships we found somewhat challenge TMT's assumption, suggesting that different defenses may dominate at different levels of death anxiety [38]. Specifically, distal defenses may be dominant in buffering death anxiety only at higher levels. Building on our research, future studies could investigate whether death anxiety has a threshold impact on other distal defenses, such as engagement in close relationships and the desire to have children [73, 74]. These endeavors have the potential to provide a more nuanced understanding of how individuals cope with anxiety related to their own mortality.

Our findings offer a novel perspective for understanding the challenges in replicating the traditional mortality salience effect on cultural worldview defense as evidenced by previous studies [32, 75–79]. Prior studies have primarily focused on only two points (i.e., low and relatively high levels) on the death anxiety continuum [28–30, 80]. For example, mortality salience paradigms typically include two conditions: mortality salience (most frequently using two open-ended questions on thinking of one's own death) and control (parallel questions about neutral or aversive topics). This approach might induce low-to-moderate levels of death anxiety in both conditions (e.g., [29]), thereby failing to drive distal defenses. Therefore, when investigating the effects of mortality salience, more than two conditions need to be included to ensure that the induced death anxiety varies from low to high levels.

The current research also has valuable practical implications. First, our findings suggest that group identification tendencies can reflect individual levels of death anxiety. A sudden increase in affiliation with social groups may indicate a moderate-to-high level of death anxiety, necessitating interventions and support systems to prevent potential psychopathological consequences. Second, our research implies that rational and experiential cognitive systems may be prominent at the low and high ends of the death anxiety spectrum, respectively. This suggests a shift in responses to escalating existential concerns. Mental health practitioners and counselors can tailor interventions to individuals' dominant processing systems to address varying levels of death anxiety and promote adaptive coping strategies.

Some methodological limitations and theoretical concerns warrant further investigation. Our focal interest was the impact of death anxiety on group identification; however, heightened group identification would presumably reduce death anxiety. Future researchers could leverage longitudinal data collected via emerging innovative collection techniques (e.g., experience sampling and ecological momentary assessment) [81] to explore bidirectional nonlinear (or linear) effects between the two variables. Alternative explanations for our findings should also be considered. For instance, previous literature has argued that close relationships function as the first distal defense, followed by self-esteem and worldview [82]. Accordingly, future research should examine whether the nonsignificant association at low-to-moderate levels of death anxiety results from prioritizing distal defenses other than group identification.

Moreover, our work inspires future exploration of the boundary conditions that affect the nonlinear association between death anxiety and group identification. One key consideration is the effect of individual health status on this relationship, which remains uncertain due to limited information on respondents' medical backgrounds in our datasets. Future studies could test our findings among individuals vulnerable to death anxiety, such as those with mental health issues or chronic illnesses. In addition, group identification is known to be influenced by various group characteristics, including perceived homogeneity and cohesion among group members [21, 83–86]. Therefore, future studies should examine whether group traits moderate the curvilinear relationship identified in our research.

## 5. Conclusion

TMT traditionally considers group identification to be effective in buffering death anxiety, and increased death anxiety is assumed to strengthen group identification. However, by adopting a nonlinear perspective, we challenge, and moreover refine, TMT. Death anxiety was not found to be associated with greater group identification until it reached a moderate-to-high level. This nonlinear association provides novel insights into the process of managing terror and merits further empirical and theoretical consideration.

## Figures and Tables

**Table 1:** Sociodemographic characteristics of the final sample in study 1.

Variable	Categories	Frequency	Percentage (%)
Gender	Male	732	52.21
	Female	670	47.79

Age (years)	18–24	77	5.49
	25–34	215	15.34
	35–44	245	17.48
	45–54	311	22.18
	55–64	303	21.61
	65+	251	17.90

Ethnicity	White British/Irish	1,235	88.09
	White non-British/Irish	68	4.85
	Indian	26	1.85
	Pakistani	13	0.93
	Chinese	15	1.07
	Afro-Caribbean	5	0.36
	African	10	0.71
	Arab	3	0.21
	Bangladeshi	4	0.29
	Other Asian	3	0.21
	Other	20	1.43

Highest educational level	Did not attend postsecondary education	549	39.16
	Postsecondary education	853	60.84

Employment	Employed	862	61.48
	Unemployed	242	17.26
	Retired	298	21.26

Household income	£0–300 per week	278	19.83
	£301–490 per week	251	17.90
	£491–740 per week	259	18.47
	£741–1111 per week	310	22.11
	£1,112 or more per week	304	21.68

Born in the UK	Yes	1,289	91.94
	No	113	8.06

Grow up in the UK	Yes	1,307	93.22
	No	95	6.78

**Table 2:** Comparisons of linear and quadratic models in studies 1 and 2.

Outcomes	Model L1 vs. Q1		Model L2 vs. Q2	
Study 1	*F* (1, 1398)	*p*	*F* (1, 1384)	*p*
Identification with my community	8.211	.004	7.187	.007
Identification with my country	8.695	.003	7.249	.007
Identification with all humans	8.357	.004	7.820	.005
Study 2	*χ* ^2^ (1)	*p*	*χ* ^2^ (1)	*p*
Perceived closeness to village, town, or city	6.408	.011	7.041	.008
Perceived closeness to county, region, or district	6.458	.011	6.798	.009
Perceived closeness to country	5.542	.019	5.352	.021
Perceived closeness to continent	11.867	<.001	7.927	.005
Perceived closeness to world	2.400	.121	1.520	.218

*Note*: models L1 and L2 are linear models without and with the covariates, respectively. Models Q1 and Q2 are quadratic models without and with the covariates, respectively.

**Table 3:** Sociodemographic characteristics of the final sample in study 2.

Variable	Categories	Frequency	Percentage (%)
Gender	Male	27,867	49.00
	Female	29,004	51.00

Age (years)	16–24	8,303	14.60
	25–34	12,488	21.96
	35–44	11,077	19.48
	45–54	9,647	16.96
	55–64	8,243	14.49
	65+	7,113	12.51

Highest educational level	Lower	16,857	29.64
	Middle	20,400	35.87
	Higher	19,614	34.49

Marital status	Married	35,658	62.70
	Divorced	2,657	4.67
	Widowed	2,994	5.26
	Never married	15,562	27.36

Employment	Employed	41,017	72.12
	Unemployed	8,112	14.26
	Retired/pensioned	7,086	12.46
	Other	656	1.15

Household income level	Low	13,209	23.23
	Medium	37,572	66.07
	High	6,090	10.71

Subjective social class	Lower	6,402	11.26
	Working	13,198	23.21
	Lower middle	23,184	40.77
	Upper middle	13,117	23.06
	Upper	970	1.71

Native citizen	I was born in this country	53,770	94.55
	I am an immigrant to this country	3,101	5.45

## Data Availability

All data and analysis scripts for both studies are available at https://osf.io/f5sh8/?view_only=75378495a64a4392a9dc65e4a5ecf42c.

## Supplementary Materials

Supplementary Materials For descriptive statistics and zero-order correlations in study 1, please see Table S1. For detailed regression results in study 1, please see Tables S2a–S2c. For detailed segmented models results in study 1, please see Table S3. For the results of the supplemental analyses in study 1, please see Tables S4–S8. For a list of countries/regions and descriptive statistics by country/region in study 2, please see Table S9. For descriptive statistics and zero-order correlations in study 2, please see Table S10. For detailed multilevel regression results in study 2, please see Tables S11a–S11e. For detailed segmented multilevel models results in study 2, please see Table S12.
