# Supplementary material for: Enough Terror to Belong: The Nonlinear Association of Death Anxiety with Group Identification
Source: Depress Anxiety. 2024 May 21;2024:3699789. doi: 10.1155/2024/3699789 (PMC11918929; doi:10.1155/2024/3699789)
Supplement: Supplementary Materials — For descriptive statistics and zero-order correlations in study 1, please see Table S1. For detailed regression results in study 1, please see Tables S2a–S2c. For detailed segmented models results in study 1, please see Table S3. For the results of the supplemental analyses in study 1, please see Tables S4–S8. For a list of countries/regions and descriptive statistics by country/region in study 2, please see Table S9. For descriptive statistics and zero-order correlations in study 2, please see Table S10. For detailed multilevel regression results in study 2, please see Tables S11a–S11e. For detailed segmented multilevel models results in study 2, please see Table S12. [file 3699789.f1.docx]

**SUPPLEMENTAL MATERIALS**

**Enough Terror to Belong: The Nonlinear Association of Death Anxiety**

**with Group Identification**

[Supplemental Results in Study 1 1](#_Toc163669857)

[Table S1 1](#_Toc163669858)

[Table S2a 2](#_Toc163669859)

[Table S2b 3](#_Toc163669860)

[Table S2c 4](#_Toc163669861)

[Table S3 5](#_Toc163669862)

[Supplemental Analyses in Study 1 6](#_Toc163669863)

[Table S4 9](#_Toc163669864)

[Table S5 10](#_Toc163669865)

[Table S6a 11](#_Toc163669866)

[Table S6b 12](#_Toc163669867)

[Table S6c 13](#_Toc163669868)

[Table S7 14](#_Toc163669869)

[Table S8 15](#_Toc163669870)

[Supplemental Results in Study 2 16](#_Toc163669871)

[Table S9 16](#_Toc163669872)

[Table S10 18](#_Toc163669873)

[Table S11a 19](#_Toc163669874)

[Table S11b 20](#_Toc163669875)

[Table S11c 21](#_Toc163669876)

[Table S11d 22](#_Toc163669877)

[Table S11e 23](#_Toc163669878)

[Table S12 24](#_Toc163669879)

# Supplemental Results in Study 1

## Table S1

*Descriptive Statistics and Zero-Order Correlations in Study 1*

| Variables | *M* | *SD* | 1 | 2 | 3 | 4 | 5 | 6 | 7 | 8 | 9 | 10 | 11 | 12 | 13 | 14 |
| --- | --- | --- | --- | --- | --- | --- | --- | --- | --- | --- | --- | --- | --- | --- | --- | --- |
| 1. Death Anxiety_wave1_ | 2.52 | 0.88 | — |  |  |  |  |  |  |  |  |  |  |  |  |  |
| 2. Identification with My Community_wave1_ | 3.38 | 0.94 | -.01 | — |  |  |  |  |  |  |  |  |  |  |  |  |
| 3. Identification with My Country_wave1_ | 3.40 | 0.85 | .00 | .83^***^ | — |  |  |  |  |  |  |  |  |  |  |  |
| 4. Identification with All Humans_wave1_ | 3.32 | 0.90 | .01 | .64^***^ | .76^***^ | — |  |  |  |  |  |  |  |  |  |  |
| 5. Identification with My Community_wave2_ | 3.36 | 0.96 | .05 | .69^***^ | .59^***^ | .48^***^ | — |  |  |  |  |  |  |  |  |  |
| 6. Identification with My Country_wave2_ | 3.33 | 0.88 | .06^*^ | .58^***^ | .64^***^ | .53^***^ | .84^***^ | — |  |  |  |  |  |  |  |  |
| 7. Identification with All Humans_wave2_ | 3.28 | 0.93 | .05 | .48^***^ | .52^***^ | .68^***^ | .65^***^ | .76^***^ | — |  |  |  |  |  |  |  |
| 8. COVID19 anxiety_wave1_ | 68.16 | 24.74 | .26^***^ | .16^***^ | .18^***^ | .16^***^ | .18^***^ | .18^***^ | .17^***^ | — |  |  |  |  |  |  |
| 9. Age_wave1_ | 49.22 | 14.94 | -.25^***^ | .08^**^ | .05 | -.08^**^ | .05 | -.00 | -.08^**^ | .05 | — |  |  |  |  |  |
| 10. Sex_wave1_ | 0.52 | 0.50 | .08^**^ | -.08^**^ | -.08^**^ | -.09^**^ | -.07^**^ | -.06^*^ | -.07^**^ | -.08^**^ | .15^***^ | — |  |  |  |  |
| 11. Highest Educational Level_wave1_ | 0.61 | 0.49 | -.06^*^ | .07^**^ | .06^*^ | .13^***^ | .06^*^ | .04 | .10^***^ | -.02 | -.07^**^ | .05 | — |  |  |  |
| 12. Born in UK_wave1_ | 0.92 | 0.27 | -.00 | -.02 | -.04 | -.09^***^ | .00 | -.01 | -.06^*^ | -.00 | .11^***^ | .04 | -.13^***^ | — |  |  |
| 13. Grow Up in UK_wave1_ | 0.93 | 0.25 | .02 | .02 | .01 | -.06^*^ | .03 | .03 | -.03 | -.01 | .08^**^ | .06^*^ | -.11^***^ | .78^***^ | — |  |
| 14. Ethnicity_wave1_ | 0.88 | 0.32 | -.07^**^ | -.02 | -.03 | -.12^***^ | -.05 | -.05 | -.13^***^ | .01 | .24^***^ | .04 | -.12^***^ | .66^***^ | .54^***^ | — |
| 15. Left-Right_wave1_ | 5.42 | 1.84 | .06^*^ | .04 | .04 | -.15^***^ | .08^**^ | .09^**^ | -.13^***^ | .02 | .13^***^ | .06^*^ | -.07^**^ | .02 | .04 | .06^*^ |

*Note*. *N* = 1,402. ^*^*p* < .05, ^**^*p* < .01, ^***^*p* < .001.

## Table S2a

*Regression Results for Identification with My Community at Wave 2 in Study 1*

| Predictors | Model L1 | Model L2 | Model Q1 | Model Q2 |
| --- | --- | --- | --- | --- |
| Death Anxiety_wave1_ | 0.055^**^ | 0.029 | 0.041^+^ | 0.017 |
|  | [0.013, 0.096] | [-0.016, 0.073] | [-0.001, 0.084] | [-0.029, 0.063] |
| Death Anxiety _wave1_: Squared |  |  | 0.058^**^ | 0.054^**^ |
|  |  |  | [0.018, 0.098] | [0.015, 0.094] |
| Identification with My Community _wave1_ | 0.702^***^ | 0.680^***^ | 0.701^***^ | 0.678^***^ |
|  | [0.663, 0.741] | [0.640, 0.719] | [0.662, 0.739] | [0.638, 0.718] |
| COVID-19 Anxiety _wave1_ |  | 0.003^***^ |  | 0.003^***^ |
|  |  | [0.001, 0.004] |  | [0.001, 0.004] |
| Age |  | -0.001 |  | -0.001 |
|  |  | [-0.004, 0.003] |  | [-0.004, 0.003] |
| Gender |  | -0.043 |  | -0.042 |
|  |  | [-0.118, 0.033] |  | [-0.118, 0.033] |
| Education |  | 0.022 |  | 0.023 |
|  |  | [-0.055, 0.100] |  | [-0.055, 0.100] |
| Employment: Unemployed |  | -0.080 |  | -0.075 |
|  |  | [-0.191, 0.032] |  | [-0.186, 0.036] |
| Employment: Retired |  | 0.044 |  | 0.050 |
|  |  | [-0.075, 0.162] |  | [-0.068, 0.168] |
| Income: £301–490 per week |  | 0.013 |  | 0.015 |
|  |  | [-0.114, 0.140] |  | [-0.111, 0.142] |
| Income: £491–740 per week |  | -0.001 |  | 0.007 |
|  |  | [-0.130, 0.127] |  | [-0.122, 0.135] |
| Income: £741–1,111 per week |  | -0.042 |  | -0.035 |
|  |  | [-0.164, 0.081] |  | [-0.157, 0.088] |
| Income: £1,112 or more per week |  | 0.089 |  | 0.098 |
|  |  | [-0.037, 0.215] |  | [-0.028, 0.224] |
| Born in the UK |  | 0.205^+^ |  | 0.188 |
|  |  | [-0.031, 0.441] |  | [-0.048, 0.423] |
| Grow Up in the UK |  | 0.072 |  | 0.071 |
|  |  | [-0.156, 0.301] |  | [-0.157, 0.299] |
| Ethnicity |  | -0.249^**^ |  | -0.237^**^ |
|  |  | [-0.402, -0.097] |  | [-0.389, -0.084] |
| Left–Right |  | 0.025^*^ |  | 0.025^*^ |
|  |  | [0.005, 0.046] |  | [0.004, 0.045] |
| (Intercept) | 0.983^***^ | 0.745^***^ | 0.943^***^ | 0.713^***^ |
|  | [0.847, 1.119] | [0.458, 1.032] | [0.805, 1.081] | [0.426, 1.000] |
| *R^2^* | .475 | .490 | .478 | .492 |
| Adjusted *R^2^* | .474 | .484 | .477 | .486 |

*Note*. *N* = 1,402. Cell entries are unstandardized estimates with 95% confidence intervals in brackets. Death anxiety was mean-centered before computing its quadratic term and entering the models. The reference category for employment: employed; for income: £0–300 per week. ^+^*p* < .10, ^*^*p* < .05, ^**^*p* < .01, ^***^*p* < .001.

## Table S2b

*Regression Results for Identification with My Country at Wave 2 in Study 1*

| Predictors | Model L1 | Model L2 | Model Q1 | Model Q2 |
| --- | --- | --- | --- | --- |
| Death Anxiety_wave1_ | 0.054^**^ | 0.020 | 0.040^+^ | 0.008 |
|  | [0.013, 0.094] | [-0.024, 0.063] | [-0.001, 0.081] | [-0.036, 0.053] |
| Death Anxiety _wave1_: Squared |  |  | 0.058^**^ | 0.053^**^ |
|  |  |  | [0.019, 0.097] | [0.014, 0.092] |
| Identification with My Country _wave1_ | 0.702^***^ | 0.680^***^ | 0.701^***^ | 0.678^***^ |
|  | [0.663, 0.741] | [0.640, 0.719] | [0.662, 0.739] | [0.638, 0.718] |
| COVID-19 Anxiety _wave1_ |  | 0.002^**^ |  | 0.002^**^ |
|  |  | [0.001, 0.004] |  | [0.001, 0.004] |
| Age |  | -0.001 |  | -0.001 |
|  |  | [-0.004, 0.002] |  | [-0.004, 0.002] |
| Gender |  | -0.031 |  | -0.031 |
|  |  | [-0.104, 0.043] |  | [-0.104, 0.043] |
| Education |  | 0.010 |  | 0.011 |
|  |  | [-0.066, 0.086] |  | [-0.065, 0.086] |
| Employment: Unemployed |  | -0.088 |  | -0.083 |
|  |  | [-0.196, 0.021] |  | [-0.192, 0.025] |
| Employment: Retired |  | -0.033 |  | -0.027 |
|  |  | [-0.149, 0.082] |  | [-0.142, 0.089] |
| Income: £301–490 per week |  | 0.065 |  | 0.067 |
|  |  | [-0.058, 0.189] |  | [-0.056, 0.191] |
| Income: £491–740 per week |  | 0.036 |  | 0.044 |
|  |  | [-0.089, 0.162] |  | [-0.081, 0.169] |
| Income: £741–1,111 per week |  | -0.036 |  | -0.029 |
|  |  | [-0.155, 0.084] |  | [-0.148, 0.090] |
| Income: £1,112 or more per week |  | 0.031 |  | 0.040 |
|  |  | [-0.092, 0.154] |  | [-0.083, 0.163] |
| Born in the UK |  | 0.150 |  | 0.132 |
|  |  | [-0.081, 0.380] |  | [-0.098, 0.362] |
| Grow Up in the UK |  | 0.101 |  | 0.100 |
|  |  | [-0.123, 0.324] |  | [-0.123, 0.323] |
| Ethnicity |  | -0.197^**^ |  | -0.184^*^ |
|  |  | [-0.346, -0.048] |  | [-0.334, -0.035] |
| Left–Right |  | 0.028^**^ |  | 0.027^**^ |
|  |  | [0.008, 0.047] |  | [0.007, 0.046] |
| (Intercept) | 1.097^***^ | 0.864^***^ | 1.063^***^ | 0.837^***^ |
|  | [0.950, 1.243] | [0.574, 1.153] | [0.915, 1.210] | [0.548, 1.127] |
| *R^2^* | .407 | .421 | .411 | .424 |
| Adjusted *R^2^* | .407 | .415 | .410 | .417 |

*Note*. *N* = 1,402. Cell entries are unstandardized estimates with 95% confidence intervals in brackets. Death anxiety was mean-centered before computing its quadratic term and entering the models. The reference category for employment: employed; for income: £0–300 per week. ^+^*p* < .10, ^*^*p* < .05, ^**^*p* < .01, ^***^*p* < .001.

## Table S2c

*Regression Results for Identification with All Humans at Wave 2 in Study 1*

| Predictors | Model L1 | Model L2 | Model Q1 | Model Q2 |
| --- | --- | --- | --- | --- |
| Death Anxiety_wave1_ | 0.042^*^ | 0.017 | 0.028 | 0.005 |
|  | [0.001, 0.083] | [-0.028, 0.062] | [-0.013, 0.070] | [-0.040, 0.050] |
| Death Anxiety _wave1_: Squared |  |  | 0.058^**^ | 0.056^**^ |
|  |  |  | [0.019, 0.098] | [0.017, 0.096] |
| Identification with All Humans _wave1_ | 0.697^***^ | 0.672^***^ | 0.694^***^ | 0.668^***^ |
|  | [0.657, 0.737] | [0.630, 0.714] | [0.654, 0.734] | [0.627, 0.710] |
| COVID-19 Anxiety _wave1_ |  | 0.002^**^ |  | 0.002^**^ |
|  |  | [0.001, 0.004] |  | [0.001, 0.004] |
| Age |  | 0.000 |  | 0.000 |
|  |  | [-0.003, 0.004] |  | [-0.003, 0.004] |
| Gender |  | -0.024 |  | -0.024 |
|  |  | [-0.099, 0.051] |  | [-0.098, 0.051] |
| Education |  | 0.030 |  | 0.031 |
|  |  | [-0.048, 0.107] |  | [-0.047, 0.108] |
| Employment: Unemployed |  | -0.039 |  | -0.035 |
|  |  | [-0.150, 0.072] |  | [-0.145, 0.076] |
| Employment: Retired |  | -0.049 |  | -0.043 |
|  |  | [-0.167, 0.068] |  | [-0.160, 0.075] |
| Income: £301–490 per week |  | 0.036 |  | 0.039 |
|  |  | [-0.090, 0.163] |  | [-0.087, 0.165] |
| Income: £491–740 per week |  | 0.073 |  | 0.081 |
|  |  | [-0.055, 0.201] |  | [-0.047, 0.209] |
| Income: £741–1,111 per week |  | -0.043 |  | -0.036 |
|  |  | [-0.165, 0.079] |  | [-0.158, 0.086] |
| Income: £1,112 or more per week |  | 0.008 |  | 0.018 |
|  |  | [-0.117, 0.134] |  | [-0.107, 0.143] |
| Born in the UK |  | 0.104 |  | 0.086 |
|  |  | [-0.131, 0.339] |  | [-0.149, 0.321] |
| Grow Up in the UK |  | 0.107 |  | 0.106 |
|  |  | [-0.120, 0.334] |  | [-0.121, 0.332] |
| Ethnicity |  | -0.220^**^ |  | -0.207^**^ |
|  |  | [-0.372, -0.067] |  | [-0.359, -0.054] |
| Left–Right |  | -0.015 |  | -0.016 |
|  |  | [-0.035, 0.006] |  | [-0.036, 0.004] |
| (Intercept) | 0.966^***^ | 0.957^***^ | 0.932^***^ | 0.932^***^ |
|  | [0.829, 1.104] | [0.651, 1.263] | [0.793, 1.071] | [0.626, 1.238] |
| *R^2^* | .458 | .467 | .461 | .470 |
| Adjusted *R^2^* | .457 | .461 | .460 | .464 |

*Note*. *N* = 1,402. Cell entries are unstandardized estimates with 95% confidence intervals in brackets. Death anxiety was mean-centered before computing its quadratic term and entering the models. The reference category for employment: employed; for income: £0–300 per week. ^+^*p* < .10, ^*^*p* < .05, ^**^*p* < .01, ^***^*p* < .001.

## Table S3

*Segmented Models Results for Each Indicator of Group Identification at Wave 2 in Study 1*

|  | Breakpoint | Slope of Segment 1 | Slope of Segment 2 |
| --- | --- | --- | --- |
| Identification with … |  |  |  |
| My Community | 3.412  [2.866, 3.957] | 0.002  [-0.057, 0.061] | 0.306  [0.072, 0.539] |
| My Country | 2.964  [2.356, 3.571] | -0.013  [-0.092, 0.065] | 0.191  [0.065, 0.317] |
| All Humans | 2.353  [1.737, 2.968] | -0.090  [-0.227, 0.046] | 0.126  [0.041, 0.211] |

*Note.* The breakpoints and slopes before (Segment 1) and after (Segment 2) the breakpoints were estimated with 95% confidence intervals in brackets by using the functions in Muggeo et al. (2008). Group identification indicators at Wave 1 were included in all the models.

# Supplemental Analyses in Study 1

In Study 1, we primarily focused on the curvilinear longitudinal effect of death anxiety on group identification by employing the first two waves of data from the C19PRC. However, we also explored the nonlinear associations between the two variables using only data from Wave 1, which comprised a nationally representative sample. After removing respondents with missing data for the key variables of interest, the final sample at Wave 1 comprised 2,021 respondents from the UK (*M*_age_ = 45.46, *SD*_age_ = 15.90, 51.81% female). Table S4 shows the sociodemographic characteristics of the Wave 1 sample. In addition, 48 respondents (2.38%) in the sample received a COVID-19 diagnosis.

The analytic strategy was almost similar to that of Study 1 in the main document, except that both the predictor and outcome variables were from Wave 1.

**Results**

Table S5 shows the descriptive statistics and zero-order correlations among the variables at Wave 1. Death anxiety was not associated with identification with one’s community (*r* = .00, *p* = .941), one’s country (*r* = .02, *p* = .347), or all humans (*r* = .03, *p* = .156). However, visual inspection of the data suggested that these associations might be curvilinear (Figure S1).


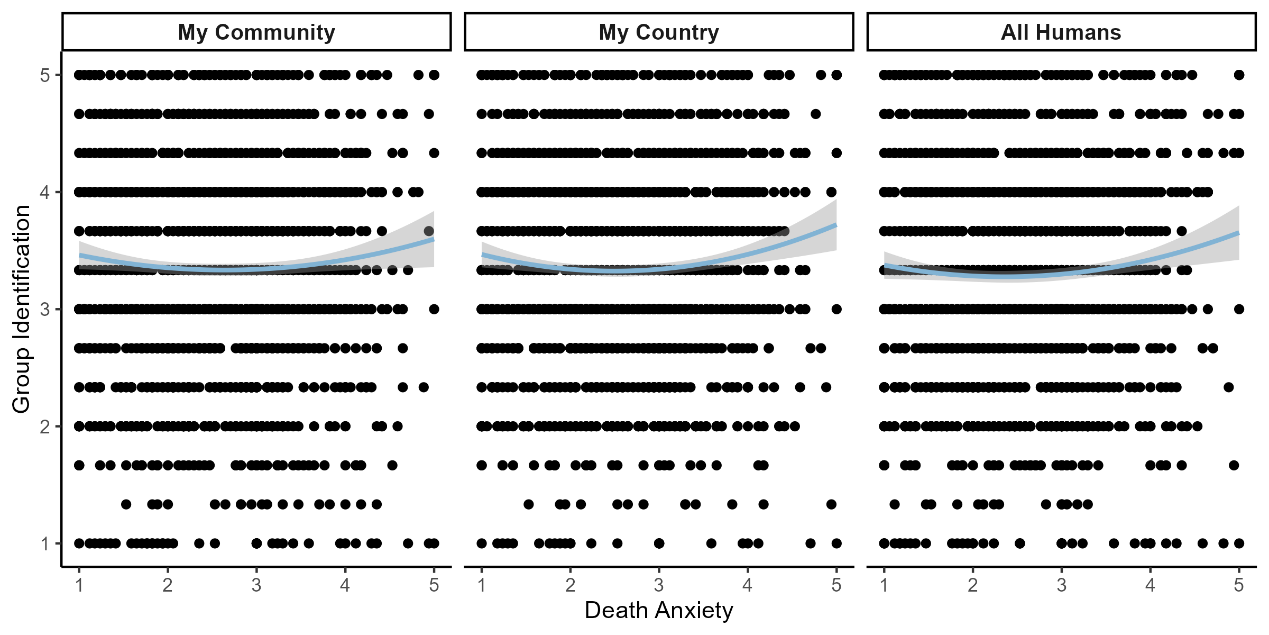


Figure S1: Death anxiety and group identification indicators at Wave 1 in Study 1. Gray shading indicates standard errors.

Next, we examined our hypothesized J-shaped relationship by conducting nonlinear association and J-shaped tests.

***Nonlinear Association*** ***Test***: ***Linear Versus Quadratic Models***

We tested the linear and quadratic models for each group identification indicator (Tables S6a–S6c). The results showed that in the linear models, both without or with the covariates, the linear terms were not significant for identification with one’s community (*p*s > .426), one’s country (*p*s > .347) or all humans (*p*s > .156). Critically, in the quadratic models for all group identification indicators, the squared terms were significantly positive (*B_community_* = 0.047, *p* = .035; *B_country_* = 0.069, *p* = .002; *B_human_* = 0.054, *p* = .013), even after controlling for the covariates (*B_community_* = 0.044, *p* = .044; *B_country_* = 0.060, *p* = .003; *B_human_* = 0.060, *p* = .005). Further, the quadratic models fit the data better than the linear models, which were robust after controlling for the covariates (Table S7). Therefore, these findings provide evidence of a curvilinear association between death anxiety and group identification and the possible presence of a J-shaped pattern.

***J-Shaped Curve Test: Segmented Models***

Results from the segmented models (Table S8) revealed a nonlinear relationship between death anxiety and group identification. Moreover, the nonlinear associations were J-shaped for identification with one’s community, and all humans. Specifically, both the breakpoints of the two indicators were 3.000; the slopes before the breakpoint (Segment 1) were nonsignificant, whereas the slopes after the breakpoint (Segment 2) were significantly positive. However, the association between death anxiety and identification with one’s country was U-shaped, with a breakpoint of 3.000, a significantly negative slope before the breakpoint, and a significantly positive slope after the breakpoint.

**Discussion**

In summary, supplemental analyses of the data at Wave 1 in Study 1 validated the curvilinear relationship: death anxiety did not show positive correlations with group identification indicators until reaching a moderate-to-high level. However, the nonlinear relationship exhibited a J-shaped pattern for identification with one’s community and all humans, whereas a U-shaped pattern was observed for identification with one’s country. Combining these results with those of Study 1 in the main text, it appears that low-to-moderate levels of death anxiety were negatively linked to national identification, but did not predict it one month later. This inconsistent finding might be due to the unique context of COVID-19. During the first wave of data collection, the number of COVID-19 cases was rapidly increasing in the UK (Boddington et al., 2020). Thus, respondents in the UK with low-to-moderate levels of death anxiety tended to reduce their death-related concerns by cognitively distancing themselves as members of their country. However, with time, they may have come to realize the inefficacy of such a strategy, leading to a lack of predictive power of low-to-moderate death anxiety levels in national identification a month later. Overall, these findings suggest the need for further investigation into the intricate association between death anxiety and group identification.

## Table S4

*Sociodemographic characteristics of the sample at Wave 1*

| Variable | Categories | Frequency | Percentage (%) |
| --- | --- | --- | --- |
| Gender | Male | 972 | 51.81 |
|  | Female | 1,047 | 48.10 |
|  | Transgender | 2 | 0.10 |
| Age (Years) | 18–24 | 246 | 12.17 |
|  | 25–34 | 378 | 18.70 |
|  | 35–44 | 352 | 17.42 |
|  | 45–54 | 410 | 20.29 |
|  | 55–64 | 348 | 17.22 |
|  | 65+ | 287 | 14.20 |
| Ethnicity | White British/Irish | 1,728 | 85.50 |
|  | White non-British/Irish | 116 | 5.74 |
|  | Indian | 41 | 2.03 |
|  | Pakistani | 27 | 1.34 |
|  | Chinese | 19 | 0.94 |
|  | Afro-Caribbean | 13 | 0.64 |
|  | African | 27 | 1.34 |
|  | Arab | 3 | 0.15 |
|  | Bangladeshi | 6 | 0.30 |
|  | Other Asian | 11 | 0.54 |
|  | Other | 30 | 1.48 |
| Highest Educational Level | Did not attend post-secondary education | 807 | 39.93 |
|  | Post-secondary education | 1,214 | 60.07 |
| Employment | Employed | 1,290 | 63.83 |
|  | Unemployed | 398 | 19.69 |
|  | Retired | 333 | 16.48 |
| Household Income | £0–300 per week | 409 | 20.24 |
|  | £301–490 per week | 409 | 20.24 |
|  | £491–740 per week | 385 | 19.05 |
|  | £741–1111 per week | 409 | 20.24 |
|  | £1,112 or more per week | 409 | 20.24 |
| Born in the UK | Yes | 1,830 | 90.55 |
|  | No | 191 | 9.45 |
| Grow up in the UK | Yes | 1,868 | 92.43 |
|  | No | 153 | 7.57 |

## Table S5

*Descriptive Statistics and Zero-Order Correlations at Wave 1 in Study 1*

| Variables | *M* | *SD* | 1 | 2 | 3 | 4 | 5 | 6 | 7 | 8 | 9 | 10 | 11 |
| --- | --- | --- | --- | --- | --- | --- | --- | --- | --- | --- | --- | --- | --- |
| 1. Death Anxiety | 2.52 | 0.88 | — |  |  |  |  |  |  |  |  |  |  |
| 2. Identification with My Community | 3.37 | 0.93 | .00 | — |  |  |  |  |  |  |  |  |  |
| 3. Identification with My Country | 3.37 | 0.85 | .02 | .82^***^ | — |  |  |  |  |  |  |  |  |
| 4. Identification with All Humans | 3.32 | 0.90 | .03 | .64^***^ | .76^***^ | — |  |  |  |  |  |  |  |
| 5. COVID19 anxiety | 67.75 | 24.58 | .26^***^ | .15^***^ | .16^***^ | .15^***^ | — |  |  |  |  |  |  |
| 6. Age | 45.46 | 15.90 | -.26^***^ | .07^**^ | .05^*^ | -.07^**^ | .06^**^ | — |  |  |  |  |  |
| 7. Sex | 0.48 | 0.51 | -.09^***^ | -.06^**^ | -.06^*^ | -.07^**^ | -.12^***^ | .20^***^ | — |  |  |  |  |
| 8. Highest Educational Level | 0.60 | 0.49 | -.08^***^ | .08^***^ | .06^**^ | .13^***^ | -.01 | -.03 | .05^*^ | — |  |  |  |
| 9. Born in UK | 0.91 | 0.29 | .00 | -.02 | -.03 | -.08^***^ | -.00 | .12^***^ | .04 | -.11^***^ | — |  |  |
| 10. Grow Up in UK | 0.92 | 0.26 | .02 | .01 | -.01 | -.07^**^ | -.01 | .08^***^ | .07^**^ | -.11^***^ | .76^***^ | — |  |
| 11. Ethnicity | 0.86 | 0.35 | -.07^**^ | -.03 | -.03 | -.12^***^ | .01 | .24^***^ | .04^*^ | -.09^***^ | .64^***^ | .53^***^ | — |
| 12. Left-Right | 5.32 | 1.86 | .07^*^ | .04 | .05^*^ | -.13^***^ | .03 | .18^***^ | .09^***^ | -.06^**^ | .04 | .05^*^ | .08^***^ |

*Note*. *N* = 2,021. ^*^*p* < .05, ^**^*p* < .01, ^***^*p* < .001.

## Table S6a

*Regression Results at Wave 1 for Identification with My Community in Study 1*

| Predictors | Model L1 | Model L2 | Model Q1 | Model Q2 |
| --- | --- | --- | --- | --- |
| Death Anxiety | 0.002 | -0.020 | -0.006 | -0.027 |
|  | [-0.045, 0.048] | [-0.070, 0.030] | [-0.053, 0.041] | [-0.077, 0.023] |
| Death Anxiety: Squared |  |  | 0.047^*^ | 0.044^*^ |
|  |  |  | [0.003, 0.092] | [0.001, 0.088] |
| COVID-19 Anxiety |  | 0.005^***^ |  | 0.005^***^ |
|  |  | [0.003, 0.007] |  | [0.003, 0.007] |
| Age |  | 0.005^*^ |  | 0.004^*^ |
|  |  | [0.001, 0.008] |  | [0.001, 0.008] |
| Gender |  | -0.160^***^ |  | -0.159^***^ |
|  |  | [-0.242, -0.078] |  | [-0.241, -0.077] |
| Education |  | 0.105^*^ |  | 0.104^*^ |
|  |  | [0.019, 0.191] |  | [0.019, 0.190] |
| Employment: Unemployed |  | -0.009 |  | -0.005 |
|  |  | [-0.124, 0.106] |  | [-0.120, 0.110] |
| Employment: Retired |  | 0.023 |  | 0.027 |
|  |  | [-0.117, 0.164] |  | [-0.113, 0.168] |
| Income: £301–490 per week |  | 0.192^**^ |  | 0.193^**^ |
|  |  | [0.060, 0.324] |  | [0.061, 0.325] |
| Income: £491–740 per week |  | 0.261^***^ |  | 0.268^***^ |
|  |  | [0.124, 0.397] |  | [0.131, 0.404] |
| Income: £741–1,111 per week |  | 0.164^*^ |  | 0.170^*^ |
|  |  | [0.030, 0.299] |  | [0.036, 0.305] |
| Income: £1,112 or more per week |  | 0.333^***^ |  | 0.337^***^ |
|  |  | [0.195, 0.470] |  | [0.199, 0.474] |
| Born in the UK |  | -0.129 |  | -0.135 |
|  |  | [-0.362, 0.104] |  | [-0.368, 0.098] |
| Grow Up in the UK |  | 0.319^**^ |  | 0.314^**^ |
|  |  | [0.085, 0.553] |  | [0.080, 0.548] |
| Ethnicity |  | -0.183^*^ |  | -0.175^*^ |
|  |  | [-0.335, -0.032] |  | [-0.326, -0.023] |
| Left–Right |  | 0.009 |  | 0.008 |
|  |  | [-0.013, 0.031] |  | [-0.015, 0.030] |
| (Intercept) | 3.371^***^ | 2.580^***^ | 3.335^***^ | 2.556^***^ |
|  | [3.330, 3.412] | [2.308, 2.851] | [3.282, 3.387] | [2.284, 2.828] |
| *R^2^* | <.001 | .056 | .002 | .058 |
| Adjusted *R^2^* | <.001 | .049 | .001 | .050 |

*Note*. *N* = 2,021. Cell entries are unstandardized estimates with 95% confidence intervals in brackets. Death anxiety was mean-centered before computing its quadratic term and entering the models. The reference category for employment: employed; for income: £0–300 per week. ^+^*p* < .10, ^*^*p* < .05, ^**^*p* < .01, ^***^*p* < .001.

## Table S6b

*Regression Results at Wave 1 for Identification with My Country in Study 1*

| Predictors | Model L1 | Model L2 | Model Q1 | Model Q2 |
| --- | --- | --- | --- | --- |
| Death Anxiety | 0.020 | -0.005 | 0.010 | -0.014 |
|  | [-0.022, 0.063] | [-0.051, 0.041] | [-0.034, 0.054] | [-0.060, 0.032] |
| Death Anxiety: Squared |  |  | 0.069^**^ | 0.060^**^ |
|  |  |  | [0.023, 0.104] | [0.021, 0.100] |
| COVID-19 Anxiety |  | 0.005^***^ |  | 0.005^***^ |
|  |  | [0.003, 0.006] |  | [0.003, 0.006] |
| Age |  | 0.003 |  | 0.003 |
|  |  | [-0.001, 0.006] |  | [-0.001, 0.006] |
| Gender |  | -0.117^**^ |  | -0.116^**^ |
|  |  | [-0.192, -0.042] |  | [-0.191, -0.041] |
| Education |  | 0.077^+^ |  | 0.076^+^ |
|  |  | [-0.002, 0.155] |  | [-0.003, 0.154] |
| Employment: Unemployed |  | -0.020 |  | -0.015 |
|  |  | [-0.125, 0.085] |  | [-0.120, 0.090] |
| Employment: Retired |  | 0.040 |  | 0.045 |
|  |  | [-0.088, 0.168] |  | [-0.083, 0.174] |
| Income: £301–490 per week |  | 0.105^+^ |  | 0.107^+^ |
|  |  | [-0.016, 0.226] |  | [-0.014, 0.228] |
| Income: £491–740 per week |  | 0.157^*^ |  | 0.167^**^ |
|  |  | [0.032, 0.283] |  | [0.042, 0.292] |
| Income: £741–1,111 per week |  | 0.051 |  | 0.059 |
|  |  | [-0.073, 0.174] |  | [-0.064, 0.182] |
| Income: £1,112 or more per week |  | 0.255^***^ |  | 0.260^***^ |
|  |  | [0.129, 0.381] |  | [0.134, 0.386] |
| Born in the UK |  | -0.157 |  | -0.166 |
|  |  | [-0.371, 0.056] |  | [-0.379, 0.047] |
| Grow Up in the UK |  | 0.211^+^ |  | 0.203^+^ |
|  |  | [-0.004, 0.425] |  | [-0.011, 0.418] |
| Ethnicity |  | -0.099 |  | -0.088 |
|  |  | [-0.238, 0.040] |  | [-0.227, 0.051] |
| Left–Right |  | 0.017 |  | 0.015 |
|  |  | [-0.004, 0.037] |  | [-0.006, 0.035] |
| (Intercept) | 3.374^***^ | 2.758^***^ | 3.326^***^ | 2.725^***^ |
|  | [3.337, 3.412] | [2.509, 3.006] | [3.278, 3.374] | [2.476, 2.974] |
| *R^2^* | <.001 | 0.050 | 0.005 | 0.054 |
| Adjusted *R^2^* | <.001 | 0.043 | 0.004 | 0.047 |

*Note*. *N* = 2,021. Cell entries are unstandardized estimates with 95% confidence intervals in brackets. Death anxiety was mean-centered before computing its quadratic term and entering the models. The reference category for employment: employed; for income: £0–300 per week. ^+^*p* < .10, ^*^*p* < .05, ^**^*p* < .01, ^***^*p* < .001.

## Table S6c

*Regression Results at Wave 1 for Identification with All Humans in Study 1*

| Predictors | Model L1 | Model L2 | Model Q1 | Model Q2 |
| --- | --- | --- | --- | --- |
| Death Anxiety | 0.032 | -0.003 | 0.024 | -0.013 |
|  | [-0.012, 0.077] | [-0.051, 0.044] | [-0.022, 0.069] | [-0.061, 0.035] |
| Death Anxiety: Squared |  |  | 0.054^*^ | 0.060^**^ |
|  |  |  | [0.011, 0.097] | [0.018, 0.101] |
| COVID-19 Anxiety |  | 0.005^***^ |  | 0.005^***^ |
|  |  | [0.004, 0.007] |  | [0.004, 0.007] |
| Age |  | -0.002 |  | -0.002 |
|  |  | [-0.005, 0.001] |  | [-0.005, 0.001] |
| Gender |  | -0.097^*^ |  | -0.097^**^ |
|  |  | [-0.176, -0.019] |  | [-0.175, -0.018] |
| Education |  | 0.176^***^ |  | 0.175^***^ |
|  |  | [0.094, 0.258] |  | [0.093, 0.257] |
| Employment: Unemployed |  | -0.055 |  | -0.049 |
|  |  | [-0.164, 0.055] |  | [-0.159, 0.060] |
| Employment: Retired |  | 0.028 |  | 0.033 |
|  |  | [-0.106, 0.162] |  | [-0.101, 0.167] |
| Income: £301–490 per week |  | 0.105 |  | 0.106^+^ |
|  |  | [-0.022, 0.232] |  | [-0.020, 0.233] |
| Income: £491–740 per week |  | 0.087 |  | 0.096 |
|  |  | [-0.043, 0.218] |  | [-0.034, 0.227] |
| Income: £741–1,111 per week |  | 0.043 |  | 0.051 |
|  |  | [-0.086, 0.172] |  | [-0.078, 0.180] |
| Income: £1,112 or more per week |  | 0.249^***^ |  | 0.255^***^ |
|  |  | [0.118, 0.381] |  | [0.123, 0.386] |
| Born in the UK |  | -0.010 |  | -0.018 |
|  |  | [-0.233, 0.214] |  | [-0.241, 0.205] |
| Grow Up in the UK |  | 0.045 |  | 0.038 |
|  |  | [-0.179, 0.270] |  | [-0.186, 0.262] |
| Ethnicity |  | -0.256^***^ |  | -0.245^***^ |
|  |  | [-0.401, -0.111] |  | [-0.390, -0.100] |
| Left–Right |  | -0.060^***^ |  | -0.062^***^ |
|  |  | [-0.081, -0.038] |  | [-0.083, -0.040] |
| (Intercept) | 3.321^***^ | 3.402^***^ | 3.279^***^ | 3.370^***^ |
|  | [3.281, 3.360] | [3.142, 3.662] | [3.228, 3.330] | [3.109, 3.630] |
| *R^2^* | .001 | 0.077 | 0.004 | 0.081 |
| Adjusted *R^2^* | .001 | 0.071 | 0.003 | 0.074 |

*Note*. *N* = 2,021. Cell entries are unstandardized estimates with 95% confidence intervals in brackets. Death anxiety was mean-centered before computing its quadratic term and entering the models. The reference category for employment: employed; for income: £0–300 per week. ^+^*p* < .10, ^*^*p* < .05, ^**^*p* < .01, ^***^*p* < .001.

## Table S7

*Comparisons of linear and quadratic models at Wave 1 in Study 1*

| Outcomes | Model L1 vs Q1 | | Model L2 vs Q2 | |
| --- | --- | --- | --- | --- |
| Wave 1 | *F* (1, 2018) | *p* | *F* (1, 2004) | *p* |
| Identification with My Community | 4.435 | .035 | 4.043 | .044 |
| Identification with My Country | 9.479 | .002 | 8.878 | .003 |
| Identification with All Humans | 6.146 | .013 | 7.948 | .005 |

*Note*. Models L1 and L2 are linear models without and with the covariates, respectively. Models Q1 and Q2 are quadratic models without and with the covariates, respectively.

## Table S8

*Segmented Models Results at Wave 1 for Each Indicator of Group Identification in Study 1*

|  | Breakpoint | Slope of Segment 1 | Slope of Segment 2 |
| --- | --- | --- | --- |
| Identification with … |  |  |  |
| My Community | 3.000  [2.454, 3.546] | -0.081  [-0.172, 0.010] | 0.173  [0.034, 0.312] |
| My Country | 3.000  [2.567, 3.433] | -0.083  [-0.155, -0.010] | 0.233  [0.086, 0.381] |
| All Humans | 3.000  [2.515, 3.485] | -0.065  [-0.142, 0.012] | 0.234  [0.078, 0.390] |

*Note.* The breakpoints and slopes before (Segment 1) and after (Segment 2) the breakpoints were estimated with 95% confidence intervals in brackets by using the functions in Muggeo et al. (2008).

# Supplemental Results in Study 2

## Table S9

*The List of Countries/Regions and Descriptive Statistics by Country/Region in Study 2*

| Country/Region | *n* | Worry About a Terrorist Attack | | Perceived Closeness to … | | | | | | | | | |
| --- | --- | --- | --- | --- | --- | --- | --- | --- | --- | --- | --- | --- | --- |
|  |  |  |  | Village, Town, or City | | County, Region, or District | | Country | | Continent | | World | |
|  |  | *M* | *SD* | *M* | *SD* | *M* | *SD* | *M* | *SD* | *M* | *SD* | *M* | *SD* |
| Andorra | 801 | 2.50 | 1.07 | 3.38 | 0.74 | 3.37 | 0.75 | 3.32 | 0.78 | 2.94 | 0.93 | 3.02 | 1.01 |
| Argentina | 708 | 2.32 | 1.09 | 3.43 | 0.62 | 3.31 | 0.68 | 3.19 | 0.74 | 2.75 | 0.83 | 2.46 | 0.91 |
| Australia | 1535 | 2.67 | 0.88 | 3.18 | 0.72 | 3.07 | 0.73 | 3.37 | 0.69 | 2.37 | 0.77 | 2.42 | 0.83 |
| Bangladesh | 1028 | 2.82 | 1.07 | 3.83 | 0.44 | 3.53 | 0.61 | 3.31 | 0.73 | 2.32 | 0.87 | 2.15 | 0.91 |
| Armenia | 605 | 3.52 | 0.73 | 3.65 | 0.57 | 3.38 | 0.68 | 3.55 | 0.57 | 2.16 | 0.82 | 2.36 | 0.88 |
| Bolivia | 1610 | 2.93 | 1.12 | 3.53 | 0.70 | 3.37 | 0.81 | 3.41 | 0.80 | 2.84 | 1.06 | 2.73 | 1.10 |
| Brazil | 940 | 2.54 | 1.17 | 3.02 | 0.80 | 2.75 | 0.90 | 2.72 | 0.91 | 2.08 | 0.93 | 2.24 | 0.96 |
| Canada | 3997 | 2.11 | 0.84 | 3.18 | 0.75 | 3.09 | 0.77 | 3.27 | 0.77 | 2.77 | 0.79 | 2.54 | 0.76 |
| Chile | 598 | 2.89 | 1.16 | 3.51 | 0.73 | 3.39 | 0.77 | 3.51 | 0.72 | 2.88 | 0.87 | 2.45 | 0.98 |
| Taiwan ROC | 1195 | 2.98 | 1.01 | 3.13 | 0.58 | 2.98 | 0.60 | 3.05 | 0.59 | 2.42 | 0.67 | 2.14 | 0.72 |
| Colombia | 1498 | 3.01 | 1.08 | 3.26 | 0.87 | 3.21 | 0.78 | 3.16 | 0.82 | 2.67 | 0.96 | 2.56 | 1.03 |
| Cyprus | 558 | 2.71 | 1.01 | 3.44 | 0.74 | 3.37 | 0.76 | 3.33 | 0.85 | 2.46 | 0.93 | 2.37 | 0.93 |
| Czechia | 1111 | 2.32 | 0.96 | 3.52 | 0.64 | 3.40 | 0.72 | 3.30 | 0.74 | 2.80 | 0.82 | 2.47 | 0.91 |
| Ecuador | 1033 | 3.09 | 0.97 | 3.25 | 0.73 | 3.20 | 0.71 | 3.17 | 0.73 | 2.66 | 0.86 | 2.53 | 0.93 |
| Ethiopia | 562 | 3.65 | 0.70 | 3.29 | 0.87 | 3.21 | 0.89 | 3.12 | 0.92 | 2.58 | 1.02 | 2.41 | 1.08 |
| Germany | 1323 | 2.68 | 0.84 | 3.31 | 0.71 | 3.30 | 0.71 | 3.35 | 0.64 | 3.06 | 0.71 | 2.70 | 0.81 |
| Greece | 923 | 2.31 | 0.97 | 3.72 | 0.54 | 3.59 | 0.63 | 3.60 | 0.60 | 2.41 | 0.98 | 2.44 | 0.97 |
| Guatemala | 1026 | 2.50 | 0.97 | 3.26 | 0.77 | 3.12 | 0.76 | 2.94 | 0.82 | 2.46 | 0.87 | 2.32 | 0.93 |
| Indonesia | 2588 | 3.66 | 0.63 | 3.19 | 0.77 | 2.62 | 0.84 | 3.00 | 0.91 | 1.94 | 0.83 | 2.36 | 1.02 |
| Japan | 764 | 3.25 | 0.84 | 3.32 | 0.71 | 3.09 | 0.67 | 3.27 | 0.66 | 2.27 | 0.75 | 1.94 | 0.75 |
| Kenya | 1073 | 3.34 | 0.93 | 3.58 | 0.71 | 3.27 | 0.77 | 3.18 | 0.80 | 2.73 | 0.91 | 2.53 | 0.99 |
| South Korea | 1245 | 2.35 | 0.81 | 3.31 | 0.63 | 3.25 | 0.66 | 3.24 | 0.67 | 2.36 | 0.71 | 2.13 | 0.70 |
| Libya | 1081 | 3.48 | 0.94 | 3.88 | 0.41 | 3.81 | 0.48 | 3.93 | 0.33 | 3.27 | 0.93 | 3.11 | 0.96 |
| Macau SAR | 761 | 1.79 | 0.87 | 3.46 | 0.65 | 3.40 | 0.65 | 2.90 | 0.74 | 2.39 | 0.72 | 2.29 | 0.73 |
| Malaysia | 1309 | 3.34 | 0.88 | 3.32 | 0.71 | 3.15 | 0.74 | 3.00 | 0.84 | 2.23 | 0.88 | 2.15 | 0.90 |
| Maldives | 923 | 3.40 | 0.80 | 3.62 | 0.64 | 3.24 | 0.79 | 3.24 | 0.77 | 2.47 | 0.86 | 2.57 | 1.00 |
| Mexico | 1477 | 3.53 | 0.91 | 3.46 | 0.75 | 3.36 | 0.79 | 3.43 | 0.80 | 2.38 | 1.10 | 2.45 | 1.17 |
| Mongolia | 1607 | 2.01 | 1.03 | 3.28 | 0.72 | 3.18 | 0.78 | 3.07 | 0.87 | 2.49 | 0.91 | 2.38 | 0.95 |
| Morocco | 1200 | 2.72 | 1.09 | 3.64 | 0.62 | 3.46 | 0.78 | 3.47 | 0.87 | 3.21 | 0.96 | 3.19 | 1.04 |
| Netherlands | 1248 | 2.15 | 0.64 | 3.12 | 0.76 | 2.67 | 0.78 | 3.00 | 0.70 | 2.44 | 0.82 | 2.20 | 0.84 |
| New Zealand | 588 | 2.23 | 0.87 | 3.13 | 0.71 | 2.95 | 0.79 | 3.36 | 0.67 | 2.48 | 0.79 | 2.42 | 0.80 |
| Nicaragua | 870 | 2.85 | 1.18 | 3.21 | 0.83 | 3.09 | 0.81 | 3.05 | 0.84 | 2.54 | 0.92 | 2.37 | 0.97 |
| Nigeria | 1109 | 3.56 | 0.81 | 3.59 | 0.70 | 3.58 | 0.66 | 3.47 | 0.67 | 3.13 | 0.85 | 3.05 | 0.90 |
| Peru | 1128 | 3.44 | 0.80 | 3.52 | 0.67 | 3.38 | 0.75 | 3.48 | 0.75 | 2.75 | 0.96 | 2.69 | 0.99 |
| Philippines | 1197 | 3.54 | 0.66 | 3.26 | 0.67 | 3.34 | 0.63 | 3.30 | 0.65 | 2.62 | 0.81 | 3.02 | 0.84 |
| Puerto Rico | 989 | 3.46 | 0.91 | 3.39 | 0.76 | 3.32 | 0.76 | 3.48 | 0.69 | 2.74 | 0.91 | 2.67 | 1.01 |
| Romania | 684 | 2.79 | 1.05 | 3.39 | 0.66 | 3.26 | 0.75 | 3.35 | 0.70 | 2.82 | 0.87 | 2.66 | 0.92 |
| Russia | 1023 | 3.20 | 0.87 | 3.33 | 0.72 | 3.08 | 0.78 | 3.05 | 0.82 | 1.93 | 0.82 | 2.08 | 0.90 |
| Serbia | 760 | 3.06 | 0.98 | 3.33 | 0.73 | 3.14 | 0.75 | 3.09 | 0.79 | 2.20 | 0.83 | 2.03 | 0.87 |
| Slovakia | 1029 | 2.47 | 0.95 | 3.58 | 0.58 | 3.51 | 0.58 | 3.45 | 0.64 | 2.86 | 0.84 | 2.54 | 0.89 |
| Zimbabwe | 1105 | 3.47 | 0.86 | 3.55 | 0.70 | 3.49 | 0.71 | 3.43 | 0.79 | 3.26 | 0.86 | 3.10 | 0.93 |
| Tajikistan | 1189 | 2.56 | 1.30 | 3.77 | 0.50 | 3.43 | 0.69 | 3.53 | 0.74 | 2.67 | 1.06 | 2.56 | 1.19 |
| Thailand | 1006 | 2.37 | 1.10 | 3.36 | 0.82 | 2.77 | 0.98 | 2.51 | 0.97 | 1.94 | 0.97 | 1.88 | 1.01 |
| Tunisia | 948 | 3.49 | 0.80 | 3.71 | 0.58 | 3.73 | 0.57 | 3.64 | 0.64 | 3.40 | 0.81 | 3.39 | 0.86 |
| Turkey | 1992 | 3.27 | 0.74 | 3.39 | 0.69 | 3.15 | 0.81 | 3.23 | 0.76 | 2.18 | 0.86 | 2.24 | 0.87 |
| Ukraine | 543 | 3.36 | 0.89 | 3.40 | 0.67 | 3.16 | 0.73 | 3.20 | 0.74 | 2.30 | 0.95 | 2.34 | 1.00 |
| United States | 2396 | 2.84 | 0.86 | 2.91 | 0.81 | 2.79 | 0.84 | 2.98 | 0.83 | 2.65 | 0.82 | 2.41 | 0.86 |
| Uruguay | 802 | 3.13 | 1.15 | 3.36 | 0.78 | 3.35 | 0.78 | 3.58 | 0.63 | 3.07 | 0.91 | 2.75 | 0.98 |
| Venezuela | 1186 | 2.71 | 1.06 | 3.50 | 0.68 | 3.39 | 0.77 | 3.41 | 0.74 | 3.03 | 0.93 | 3.00 | 0.97 |

## Table S10

*Descriptive Statistics and Zero-Order Correlations in Study 2*

| Variables | *M* | *SD* | 1 | 2 | 3 | 4 | 5 | 6 | 7 | 8 | 9 | 10 | 11 |
| --- | --- | --- | --- | --- | --- | --- | --- | --- | --- | --- | --- | --- | --- |
| 1. Worry about a Terrorist Attack | 2.88 | 1.06 | — |  |  |  |  |  |  |  |  |  |  |
| 2. Perceived Closeness to Village, Town or City | 3.37 | 0.74 | .08^***^ | — |  |  |  |  |  |  |  |  |  |
| 3. Perceived Closeness to County, Region, or District | 3.21 | 0.80 | .07^***^ | .65^***^ | — |  |  |  |  |  |  |  |  |
| 4. Perceived Closeness to Country | 3.25 | 0.80 | .09^***^ | .46^***^ | .58^***^ | — |  |  |  |  |  |  |  |
| 5. Perceived Closeness to Continent | 2.59 | 0.94 | .02^***^ | .26^***^ | .39^***^ | .48^***^ | — |  |  |  |  |  |  |
| 6. Perceived Closeness to World | 2.51 | 0.98 | .06^***^ | .21^***^ | .31^***^ | .41^***^ | .68^***^ | — |  |  |  |  |  |
| 7. Age | 43.10 | 16.54 | -.04^***^ | .04^***^ | .05^***^ | .10^***^ | .01^**^ | -.02^***^ | — |  |  |  |  |
| 8. Sex | 0.49 | 0.50 | -.05^***^ | .00 | .00 | .02^***^ | .03^***^ | .01^*^ | .03^***^ | — |  |  |  |
| 9. Household Income | 5.01 | 2.08 | -.10^***^ | .02^***^ | .03^***^ | .04^***^ | .08^***^ | .05^***^ | -.08^***^ | .03^***^ | — |  |  |
| 10. Subjective Social Status | 2.81 | 0.97 | -.10^***^ | 0.01 | .02^***^ | .03^***^ | .08^***^ | .05^***^ | -.02^***^ | .01^***^ | .49^***^ | — |  |
| 11. Native Citizen | 0.95 | 0.23 | .09^***^ | .07^***^ | .04^***^ | .02^***^ | -.02^***^ | -.03^***^ | -.06^***^ | -.00 | -.02^***^ | -.03^***^ | — |
| 12. Left-Right | 5.71 | 2.48 | .07^***^ | .07^***^ | .06^***^ | .05^***^ | -.00 | .02^***^ | -.01 | .02^***^ | .06^***^ | .01 | .05^***^ |

*Note*. *N* = 56,871. Number of countries = 49. ^*^*p* < .05, ^**^*p* < .01, ^***^*p* < .001.

## Table S11a

*Multilevel Regression Results for Perceived Closeness to Village, Town or City in Study 2*

| Predictors | Model L1 | Model L2 | Model Q1 | Model Q2 |
| --- | --- | --- | --- | --- |
| Fixed Effects |  |  |  |  |
| Worry About a Terrorist Attack | 0.040^***^ | 0.039^***^ | 0.043^***^ | 0.042^***^ |
|  | [0.034, 0.047] | [0.033, 0.045] | [0.037, 0.050] | [0.036, 0.049] |
| Worry About a Terrorist Attack: Squared |  |  | 0.008^*^ | 0.008^**^ |
|  |  |  | [0.002, 0.013] | [0.002, 0.014] |
| Covariates |  |  |  |  |
| Age |  | 0.003^***^ |  | 0.003^***^ |
|  |  | [0.002, 0.003] |  | [0.002, 0.003] |
| Gender |  | 0.007 |  | 0.007 |
|  |  | [-0.005, 0.019] |  | [-0.005, 0.019] |
| Education: Middle |  | -0.019^*^ |  | -0.019^*^ |
|  |  | [-0.035, -0.003] |  | [-0.035, -0.003] |
| Education: Higher |  | -0.009 |  | -0.009 |
|  |  | [-0.027, 0.008] |  | [-0.026, 0.008] |
| Marital status: Divorced |  | -0.056^***^ |  | -0.057^***^ |
|  |  | [-0.085, -0.028] |  | [-0.085, -0.029] |
| Marital status: Widowed |  | -0.003 |  | -0.004 |
|  |  | [-0.032, 0.025] |  | [-0.032, 0.024] |
| Marital status: Never Married |  | -0.025^**^ |  | -0.025^**^ |
|  |  | [-0.041, -0.010] |  | [-0.041, -0.010] |
| Employment: Unemployed |  | -0.016^+^ |  | -0.016^+^ |
|  |  | [-0.034, 0.003] |  | [-0.034, 0.002] |
| Employment: Retired/Pensioned |  | 0.019^+^ |  | 0.019^+^ |
|  |  | [-0.003, 0.041] |  | [-0.004, 0.041] |
| Employment: Other |  | -0.034 |  | -0.035 |
|  |  | [-0.089, 0.021] |  | [-0.090, 0.020] |
| Household Income |  | 0.007^***^ |  | 0.007^***^ |
|  |  | [0.004, 0.011] |  | [0.004, 0.011] |
| Subjective Social Class |  | 0.013^***^ |  | 0.014^***^ |
|  |  | [0.006, 0.021] |  | [0.006, 0.021] |
| Native Citizen |  | 0.162^***^ |  | 0.163^***^ |
|  |  | [0.133, 0.191] |  | [0.134, 0.192] |
| Left–Right |  | 0.011^***^ |  | 0.010^***^ |
|  |  | [0.008, 0.013] |  | [0.008, 0.013] |
| (Intercept) | 3.402^***^ | 3.011^***^ | 3.395^***^ | 3.003^***^ |
|  | [3.343, 3.461] | [2.938, 3.085] | [3.336, 3.455] | [2.930, 3.077] |
| Random Effects |  |  |  |  |
| Var (country) | .042 | .043 | .042 | .042 |

*Note*. *N* = 56,871. Number of countries = 49. Cell entries are unstandardized estimates with 95% confidence intervals in brackets. Worry about a terrorist attack was group mean-centered before computing its quadratic term and entering the models. The reference category for education: lower; for marital status: married; for employment: employed. ^+^*p* < .10, ^*^*p* < .05, ^**^*p* < .01, ^***^*p* < .001.

## Table S11b

*Multilevel Regression Results for Perceived Closeness to County, Region, or District in Study 2*

| Predictors | Model L1 | Model L2 | Model Q1 | Model Q2 |
| --- | --- | --- | --- | --- |
| Fixed Effects |  |  |  |  |
| Worry About a Terrorist Attack | 0.050^***^ | 0.048^***^ | 0.053^***^ | 0.052^***^ |
|  | [0.044, 0.057] | [0.042, 0.055] | [0.046, 0.060] | [0.045, 0.059] |
| Worry About a Terrorist Attack: Squared |  |  | 0.008^*^ | 0.008^**^ |
|  |  |  | [0.002, 0.014] | [0.002, 0.014] |
| Covariates |  |  |  |  |
| Age |  | 0.003^***^ |  | 0.003^***^ |
|  |  | [0.003, 0.004] |  | [0.003, 0.004] |
| Gender |  | 0.007 |  | 0.007 |
|  |  | [-0.005, 0.020] |  | [-0.005, 0.020] |
| Education: Middle |  | 0.003 |  | 0.004 |
|  |  | [-0.013, 0.020] |  | [-0.013, 0.021] |
| Education: Higher |  | -0.001 |  | -0.001 |
|  |  | [-0.019, 0.018] |  | [-0.019, 0.018] |
| Marital status: Divorced |  | -0.063^***^ |  | -0.064^***^ |
|  |  | [-0.093, -0.034] |  | [-0.094, -0.034] |
| Marital status: Widowed |  | -0.019 |  | -0.019 |
|  |  | [-0.049, 0.011] |  | [-0.049, 0.011] |
| Marital status: Never Married |  | -0.022^**^ |  | -0.022^**^ |
|  |  | [-0.038, -0.005] |  | [-0.038, -0.005] |
| Employment: Unemployed |  | -0.043^***^ |  | -0.043^***^ |
|  |  | [-0.062, -0.023] |  | [-0.062, -0.023] |
| Employment: Retired/Pensioned |  | 0.02 |  | 0.02 |
|  |  | [-0.004, 0.043] |  | [-0.004, 0.043] |
| Employment: Other |  | -0.038 |  | -0.038 |
|  |  | [-0.096, 0.020] |  | [-0.097, 0.020] |
| Household Income |  | 0.010^***^ |  | 0.01^***^ |
|  |  | [0.006, 0.013] |  | [0.006, 0.013] |
| Subjective Social Class |  | 0.012^**^ |  | 0.012^**^ |
|  |  | [0.004, 0.019] |  | [0.004, 0.019] |
| Native Citizen |  | 0.139^***^ |  | 0.139^***^ |
|  |  | [0.108, 0.169] |  | [0.109, 0.170] |
| Left–Right |  | 0.015^***^ |  | 0.015^***^ |
|  |  | [0.013, 0.018] |  | [0.012, 0.018] |
| (Intercept) | 3.246^***^ | 2.817^***^ | 3.239^***^ | 2.809 ^***^ |
|  | [3.173, 3.318] | [2.730, 2.904] | [3.166, 3.311] | [2.l722, 2.896] |
| Random Effects |  |  |  |  |
| Var (country) | .063 | .066 | .063 | .065 |

*Note*. *N* = 56,871. Number of countries = 49. Cell entries are unstandardized estimates with 95% confidence intervals in brackets. Worry about a terrorist attack was group mean-centered before computing its quadratic term and entering the models. The reference category for education: lower; for marital status: married; for employment: employed. ^+^*p* < .10, ^*^*p* < .05, ^**^*p* < .01, ^***^*p* < .001.

## Table S11c

*Multilevel Regression Results for Perceived Closeness to Country in Study 2*

| Predictors | Model L1 | Model L2 | Model Q1 | Model Q2 |
| --- | --- | --- | --- | --- |
| Fixed Effects |  |  |  |  |
| Worry About a Terrorist Attack | 0.052^***^ | 0.051^***^ | 0.055^***^ | 0.054^***^ |
|  | [0.045, 0.058] | [0.044, 0.058] | [0.047, 0.062] | [0.047, 0.061] |
| Worry About a Terrorist Attack: Squared |  |  | 0.008^*^ | 0.007^*^ |
|  |  |  | [0.001, 0.014] | [0.001, 0.014] |
| Covariates |  |  |  |  |
| Age |  | 0.005^***^ |  | 0.005^***^ |
|  |  | [0.004, 0.005] |  | [0.004, 0.005] |
| Gender |  | 0.037^***^ |  | 0.037^***^ |
|  |  | [0.024, 0.050] |  | [0.024, 0.050] |
| Education: Middle |  | 0.026^**^ |  | 0.026^**^ |
|  |  | [0.009, 0.043] |  | [0.009, 0.043] |
| Education: Higher |  | 0.037^***^ |  | 0.037^***^ |
|  |  | [0.018, 0.055] |  | [0.018, 0.056] |
| Marital status: Divorced |  | -0.007 |  | -0.007 |
|  |  | [-0.037, 0.024] |  | [-0.037, 0.023] |
| Marital status: Widowed |  | -0.015 |  | -0.015 |
|  |  | [-0.045, 0.015] |  | [-0.045, 0.015] |
| Marital status: Never Married |  | -0.012 |  | -0.013 |
|  |  | [-0.029, 0.004] |  | [-0.029, 0.004] |
| Employment: Unemployed |  | -0.008 |  | -0.008 |
|  |  | [-0.028, 0.011] |  | [-0.028, 0.011] |
| Employment: Retired/Pensioned |  | 0.032^**^ |  | 0.032^**^ |
|  |  | [ 0.008, 0.055] |  | [ 0.008, 0.055] |
| Employment: Other |  | -0.063^*^ |  | -0.063^*^ |
|  |  | [-0.122, -0.004] |  | [-0.122, -0.004] |
| Household Income |  | 0.011^***^ |  | 0.011^***^ |
|  |  | [0.008, 0.015] |  | [0.008, 0.015] |
| Subjective Social Class |  | 0.012^**^ |  | 0.012^**^ |
|  |  | [0.004, 0.019] |  | [0.004, 0.019] |
| Native Citizen |  | 0.083^***^ |  | 0.084^***^ |
|  |  | [0.052, 0.114] |  | [0.053, 0.115] |
| Left–Right |  | 0.015^***^ |  | 0.015^***^ |
|  |  | [0.012, 0.017] |  | [0.012, 0.017] |
| (Intercept) | 3.266^***^ | 2.776 ^***^ | 3.260^***^ | 2.769 ^***^ |
|  | [3.194, 3.339] | [2.692, 2.861] | [3.187, 3.332] | [2.684, 2.854] |
| Random Effects |  |  |  |  |
| Var (country) | .063 | .060 | .063 | .060 |

*Note*. *N* = 56,871. Number of countries = 49. Cell entries are unstandardized estimates with 95% confidence intervals in brackets. Worry about a terrorist attack was group mean-centered before computing its quadratic term and entering the models. The reference category for education: lower; for marital status: married; for employment: employed. ^+^*p* < .10, ^*^*p* < .05, ^**^*p* < .01, ^***^*p* < .001.

## Table S11d

*Multilevel Regression Results for Perceived Closeness to Continent in Study 2*

| Predictors | Model L1 | Model L2 | Model Q1 | Model Q2 |
| --- | --- | --- | --- | --- |
| Fixed Effects |  |  |  |  |
| Worry About a Terrorist Attack | 0.038^***^ | 0.044^***^ | 0.033^***^ | 0.040^***^ |
|  | [0.030, 0.045] | [0.036, 0.051] | [0.024, 0.041] | [0.031, 0.048] |
| Worry About a Terrorist Attack: Squared |  |  | -0.013^***^ | -0.010^**^ |
|  |  |  | [-0.020, -0.005] | [-0.018, -0.003] |
| Covariates |  |  |  |  |
| Age |  | 0.003^***^ |  | 0.003^***^ |
|  |  | [0.002, 0.003] |  | [0.002, 0.003] |
| Gender |  | 0.048^***^ |  | 0.048^***^ |
|  |  | [0.033, 0.062] |  | [0.033, 0.063] |
| Education: Middle |  | 0.072^***^ |  | 0.071^***^ |
|  |  | [0.052, 0.091] |  | [0.051, 0.091] |
| Education: Higher |  | 0.133^***^ |  | 0.133^***^ |
|  |  | [0.112, 0.155] |  | [0.111, 0.155] |
| Marital status: Divorced |  | 0.002 |  | 0.002 |
|  |  | [-0.033, 0.037] |  | [-0.033, 0.037] |
| Marital status: Widowed |  | -0.029 |  | -0.028 |
|  |  | [-0.064, 0.006] |  | [-0.063, 0.006] |
| Marital status: Never Married |  | 0.015 |  | 0.015 |
|  |  | [-0.004, 0.033] |  | [-0.004, 0.034] |
| Employment: Unemployed |  | -0.018 |  | -0.018 |
|  |  | [-0.041, 0.004] |  | [-0.041, 0.005] |
| Employment: Retired/Pensioned |  | -0.009 |  | -0.009 |
|  |  | [-0.036, 0.019] |  | [-0.036, 0.019] |
| Employment: Other |  | -0.003 |  | -0.002 |
|  |  | [-0.071, 0.065] |  | [-0.071, 0.066] |
| Household Income |  | 0.020^***^ |  | 0.020^***^ |
|  |  | [0.016, 0.024] |  | [0.016, 0.024] |
| Subjective Social Class |  | 0.049^***^ |  | 0.049^***^ |
|  |  | [0.040, 0.058] |  | [0.040, 0.058] |
| Native Citizen |  | 0.015 |  | 0.014 |
|  |  | [-0.021, 0.051] |  | [-0.022, 0.050] |
| Left–Right |  | 0.005^**^ |  | 0.005^**^ |
|  |  | [0.002, 0.008] |  | [0.002, 0.008] |
| (Intercept) | 2.599^***^ | 2.116^***^ | 2.611^***^ | 2.126^***^ |
|  | [2.496, 2.703] | [1.997, 2.234] | [2.507, 2.715] | [2.008, 2.245] |
| Random Effects |  |  |  |  |
| Var (country) | .129 | .135 | .130 | .135 |

*Note*. *N* = 56,871. Number of countries = 49. Cell entries are unstandardized estimates with 95% confidence intervals in brackets. Worry about a terrorist attack was group mean-centered before computing its quadratic term and entering the models. The reference category for education: lower; for marital status: married; for employment: employed. ^+^*p* < .10, ^*^*p* < .05, ^**^*p* < .01, ^***^*p* < .001.

## Table S11e

*Multilevel Regression Results for Perceived Closeness to World in Study 2*

| Predictors | Model L1 | Model L2 | Model Q1 | Model Q2 |
| --- | --- | --- | --- | --- |
| Fixed Effects |  |  |  |  |
| Worry About a Terrorist Attack | 0.034^***^ | 0.038^***^ | 0.031^***^ | 0.036^***^ |
|  | [0.025, 0.042] | [0.030, 0.046] | [0.022, 0.040] | [0.027, 0.045] |
| Worry About a Terrorist Attack: Squared |  |  | -0.006 | -0.005 |
|  |  |  | [-0.014, 0.002] | [-0.012, 0.003] |
| Covariates |  |  |  |  |
| Age |  | 0.001^***^ |  | 0.001^***^ |
|  |  | [0.001, 0.002] |  | [0.001, 0.002] |
| Gender |  | 0.010 |  | 0.010 |
|  |  | [-0.006, 0.025] |  | [-0.006, 0.025] |
| Education: Middle |  | -0.025^*^ |  | -0.025^*^ |
|  |  | [-0.046, -0.004] |  | [-0.046, -0.004] |
| Education: Higher |  | 0.035^**^ |  | 0.035^**^ |
|  |  | [0.012, 0.058] |  | [0.012, 0.058] |
| Marital status: Divorced |  | -0.001 |  | -0.001 |
|  |  | [-0.039, 0.036] |  | [-0.038, 0.036] |
| Marital status: Widowed |  | -0.012 |  | -0.012 |
|  |  | [-0.049, 0.025] |  | [-0.049, 0.025] |
| Marital status: Never Married |  | 0.025^*^ |  | 0.025^*^ |
|  |  | [0.005, 0.045] |  | [0.005, 0.045] |
| Employment: Unemployed |  | 0.012 |  | 0.012 |
|  |  | [-0.012, 0.036] |  | [-0.012, 0.036] |
| Employment: Retired/Pensioned |  | -0.030^*^ |  | -0.030^*^ |
|  |  | [-0.059, -0.001] |  | [-0.059, -0.001] |
| Employment: Other |  | -0.059 |  | -0.059 |
|  |  | [-0.131, 0.014] |  | [-0.131, 0.014] |
| Household Income |  | 0.017^***^ |  | 0.017^***^ |
|  |  | [0.013, 0.022] |  | [0.013, 0.022] |
| Subjective Social Class |  | 0.054^***^ |  | 0.054^***^ |
|  |  | [0.044, 0.063] |  | [0.044, 0.063] |
| Native Citizen |  | -0.091^***^ |  | -0.091^***^ |
|  |  | [-0.129, -0.053] |  | [-0.129, -0.053] |
| Left–Right |  | 0.004^**^ |  | 0.004^**^ |
|  |  | [0.001, 0.007] |  | [0.001, 0.007] |
| (Intercept) | 2.507^***^ | 2.259^***^ | 2.512^***^ | 2.264 ^***^ |
|  | [2.410, 2.603] | [2.146, 2.373] | [2.416, 2.609] | [2.151, 2.378] |
| Random Effects |  |  |  |  |
| Var (country) | .112 | .116 | .112 | .116 |

*Note*. *N* = 56,871. Number of countries = 49. Cell entries are unstandardized estimates with 95% confidence intervals in brackets. Worry about a terrorist attack was group mean-centered before computing its quadratic term and entering the models. The reference category for education: lower; for marital status: married; for employment: employed. ^+^*p* < .10, ^*^*p* < .05, ^**^*p* < .01, ^***^*p* < .001.

## Table S12

*Segmented Multilevel Models Results for Each Indicator of Group Identification in Study 2*

|  | Breakpoint | Slope of Segment 1 | Slope of Segment 2 |
| --- | --- | --- | --- |
| Perceived Closeness to … |  |  |  |
| Village, Town or City | 2.534  [2.304, 2.765] | -0.002  [-0.043, 0.004] | 0.092  [0.065, 0.119] |
| County, Region, or District | 2.576  [2.339, 2.814] | -0.008  [-0.035, 0.020] | 0.101  [0.065, 0.137] |
| Country | 2.679  [2.438, 2.919] | -0.002  [-0.030, 0.025] | 0.106  [0.069, 0.142] |
| Continent | 2.401  [2.138, 2.663] | 0.039  [0.007, 0.072] | 0.028  [-0.021, 0.077] |
| World | 2.686  [2.396, 2.976] | 0.010  [-0.025, 0.046] | 0.041  [-0.011, 0.094] |

*Note.* The breakpoints and slopes before (Segment 1) and after (Segment 2) the breakpoints were estimated with 95% confidence intervals in brackets by using the functions in Muggeo et al. (2014).
